# Breast cancer and combined oral contraceptives: results from a multinational study. The WHO Collaborative Study of Neoplasia and Steroid Contraceptives.

**DOI:** 10.1038/bjc.1990.23

**Published:** 1990-01

**Authors:** 

## Abstract

A collaborative, hospital-based case-control study was conducted at 12 participating centres in 10 countries. Based on data from personal interviews of 2,116 women with newly diagnosed breast cancer and 12,077 controls, the relative risk of breast cancer in women who ever used oral contraceptives was estimated to be 1.15 (1.02, 1.29). Estimated values of this relative risk based on data from three developed and seven developing countries were 1.07 (0.91, 1.26) and 1.24 (1.05, 1.47) respectively; these estimates are not significantly different (P = 0.22). Estimates for women under and over age 35 were 1.26 (0.95, 1.66) and 1.12 (0.98, 1.27), respectively, and these estimates are also not significantly different (P = 0.38). Risk was highest in recent and current users and declined with time since last use regardless of use. Risk did not increase with duration of use after stratifying on time since last use. Risk did not increase significantly with increasing duration of use before age 25 or before a first live birth. However, a relative risk of 1.5 that was of borderline statistical significance was observed in women who used oral contraceptives for more than 2 years before age 25. No single source of bias or confounding was identified that could explain the small increases in risk that were observed. Chance alone is also an unlikely explanation. The results could be due to a combination of chance and potential sources of bias, or they could represent a weak causal relationship.


Br. J. Cancer (1990) 61, 110-119 © Macmillan Press Ltd., 1990

### Breast cancer and combined oral contraceptives: results from a multinational study

The WHO Collaborative Study of Neoplasia and Steroid Contraceptives*

**Summary** A collaborative, hospital-based case-control study was conducted at 12 participating centres in 10 countries. Based on data from personal interviews of 2,116 women with newly diagnosed breast cancer and 12,077 controls, the relative risk of breast cancer in women who ever used oral contraceptives was estimated to be 1.15 (1.02, 1.29). Estimated values of this relative risk based on data from three developed and seven developing countries were 1.07 (0.91, 1.26) and 1.24 (1.05, 1.47) respectively; these estimates are not significantly different ($P = 0.22$). Estimates for women under and over age 35 were 1.26 (0.95, 1.66) and 1.12 (0.98, 1.27), respectively, and these estimates are also not significantly different ($P = 0.38$). Risk was highest in recent and current users and declined with time since last use regardless of duration of use. Risk did not increase with duration of use after stratifying on time since last use. Risk did not increase significantly with increasing duration of use before age 25 or before a first live birth. However, a relative risk of 1.5 that was of borderline statistical significance was observed in women who used oral contraceptives for more than 2 years before age 25. No single source of bias or confounding was identified that could explain the small increases in risk that were observed. Chance alone is also an unlikely explanation. The results could be due to a combination of chance and potential sources of bias, or they could represent a weak causal relationship.

The results of studies of oral contraceptives and breast cancer have recently been reviewed and quantitatively summarised (Prentice & Thomas, 1987; Thomas, 1988). Sixteen case-control and four cohort studies all found no significant increase in risk of breast cancer in women who have ever taken oral contraceptives. Of six case-control studies that assessed risk in users of more than 10 years' duration, four found no increase in risk in such users, and although one found a significantly increased risk, another found a significant reduction in risk. Risk of breast cancer was not found to be altered from 10 to more than 20 years after initial use of oral contraceptives in eight case-control and two cohort studies.

Despite these consistent and reassuring findings, there remain legitimate concerns that oral contraceptives may enhance risk of breast cancer under some circumstances of use. Findings from 14 studies that have assessed risk of breast cancer in women who used oral contraceptives before their first live birth or full-term pregnancy are inconsistent, with eight showing no significant elevation in risk associated with such use (Vessey *et al.*, 1982; Hennekens *et al.*, 1984; Stadel *et al.*, 1985; Lipnick *et al.*, 1986; Meirik *et al.*, 1986; Paul *et al.*, 1986; Miller *et al.*, 1986; Jick *et al.*, 1989), four finding a significant trend of increasing risk with months of use before the woman's first birth (Paffenbarger *et al.*, 1980; Pike *et al.*, 1981; Harris *et al.*, 1982; McPherson *et al.*, 1987) and two (Miller *et al.*, 1989; UK National Case-Control Study Group, 1989) finding an increased risk in young women who used oral contraceptives both before and after their first term pregnancy. Three case-control studies have also found an increase in risk in women who first used oral contraceptives before age 25 (Pike *et al.*, 1983; Olsson *et al.*, 1985; Meirik *et al.*, 1986), although four others have not (Paul *et al.*, 1986; Cancer and Steroid Hormone Study (CASH), 1986; Miller *et al.*, 1986, 1989). Since the reasons for these discrepant findings are unknown, the influence of oral contraceptives when used early in a woman's reproductive life on subsequent risk of breast cancer requires further evaluation.

Another unresolved concern is whether women who are at increased risk of breast cancer should take oral contraceptives. Studies have not shown oral contraceptives to be associated with an increase in risk of breast cancer in women who are nulliparous, had their first child late in reproductive life, or had a family history of breast cancer (Thomas, 1988). Most studies have also not found an increase in risk in relation to oral contraceptive use in women with a prior history of benign breast disease (Kelsey *et al.*, 1978; Brinton *et al.*, 1982; Vessey *et al.*, 1983; Rosenberg *et al.*, 1984; CASH, 1986), including the two that specifically assessed risk in women who used oral contraceptives after a benign breast lesion developed (Brinton *et al.*, 1982; CASH, 1986). Lees *et al.* (1978), however, did find an increasing risk with duration of use in women with prior benign lesions and Paffenbarger *et al.* (1980) reported a similar result, but with a less striking trend, in premenopausal women only; Miller *et al.* (1989) also found an increase in risk in users with prior cystic disease. Pike *et al.* (1981) reported risk in relation to use before a woman's first term pregnancy to be particularly enhanced in women with a history of benign breast disease. These findings require confirmation. Also, the possible influence of oral contraceptives on risk of breast cancer in women with and without other risk factors for breast cancer should be evaluated.

Finally, almost all prior studies of breast cancer have been conducted in economically developed countries with relatively high incidence rates of this disease, and it is not certain that results from such studies are applicable to less developed countries where rates of breast cancer tend to be lower, and where the primary determinants of risk and patterns of use of oral contraceptives may be different from those in more developed countries. The WHO Collaborative Study of Neoplasia and Steroid Contraceptives was conducted, in part, to determine whether findings from studies of oral contraceptives and cancer in developed countries are similar to those from less developed parts of the world. This paper is a report of results from countries with varying levels of economic development that addresses these outstanding issues regarding combined oral contraceptives and breast cancer.

### Methods

The methods used in this study have been previously described (WHO Collaborative Study of Neoplasia and Steroid Contraceptives, 1985). Data were collected in 12 participating centres in Australia, Chile, the People's Republic of China, Colombia, the German Democratic Republic (GDR), Israel, Kenya, Mexico, the Phillippines and Thailand. Data were collected from three separate centres in Thailand (Siriraj and Chulalongkorn in Bangkok, and Chiang Mai). Some centres

Prepared by D.B. Thomas & E.A. Noonan.
*Participating investigators are listed at the end of this paper.
Correspondence: D.B.Thomas, Fred Hutchinson Cancer Research Center, 1124 Columbia Street, Seattle, WA 98104, USA.
Received 17 August 1988; and in revised form 21 June 1989.

were individual hospitals; in others, data were collected from more than one hospital. Data collection began between October 1979 and November 1982, depending on the centre, and ceased in September 1984, except in the GDR, China, Thailand, Kenya and Mexico, where data collection continued past the time the analyses for this report began. This report is based on data from cases and controls with complete information at the co-ordinating centre as of 14 February 1986.

In each hospital, cases were detected by monitoring all new admissions to wards where women with breast cancer were treated, and by checking outpatient gynaecological and tumour clinics, and records of hospital pathology laboratories. Cases included all women diagnosed locally as having a malignant breast tumour, born either after 1924 or after 1929 (depending on when oral contraceptives were first locally available), and who resided during the preceding year in a defined geographical area served by the hospital.

Controls were selected from among women admitted to other than obstetric and gynaecological wards, who met the same age and residential criteria for eligibility as the cases, and who were not admitted for treatment of conditions considered *a priori* possibly to alter contraceptive practices (i.e. circulatory and cardiovascular diseases, diabetes, chronic renal disease, benign breast disease, a previously diagnosed malignancy, chronic liver disease, and any obstetrical or gynaecological condition).

Approximately two controls were selected for each case, but controls were not matched to individual cases. A list of wards from which controls were to be selected was developed for each hospital. Each week, wards were visited in the order listed. At the time of a visit, all women eligible as controls who were admitted to the ward within the past 24 hours were selected as controls. The next ward on the list was then visited, and this procedure was repeated until sufficient controls were selected to give a cumulative ratio of two controls per case from the hospital. The same procedure was followed in the next week, beginning with the ward listed after the last one visited. This method resulted in a disproportionate number of young controls, and after the first year of the study it was modified so that during a fixed number of weeks in each month (the number varying depending on the hospital), only women in the older eligible age groups were selected. As this was a study of cancers in addition to those of the breast (i.e. cervix and corpus uteri, ovary and liver), more than two controls per breast cancer case were available for analysis.

A standardised questionnaire was administered to all study subjects by specially trained female interviewers to obtain information on the known and suspected risk factors for the neoplasms under study, and a complete obstetric and contraceptive history. Nearly all interviews were conducted in hospitals. A calendar and samples of locally available oral contraceptives were used to facilitate recall of times of use and products taken. In addition, the medical records of women who gave a history of oral contraceptive use were reviewed when available, and in such instances information from both interviews and these records were utilised by the interviewers to record details of the women's use. The questionnaire was printed in the local language in all countries except Kenya, where multiple languages are spoken, and the Philippines, where English is widely used. Where the information was not recorded directly on the English version questionnaire, it was transcribed on to an English version for mailing to the co-ordinating centre in Seattle.

Pathologists at each centre were responsible for provisionally diagnosing the cases, providing information on the extent of disease at diagnosis and gross pathology, and preparing stained histological slides for review. Slides from all cases were sent to a single reference pathologist for confirmation of diagnosis and uniform histological classification according to the WHO histological classification of breast tumours (World Health Organization, 1981).

The questionnaires, and forms from the local and reference pathologists, were key entered and edited at the co-ordinating centre. Errors that could not be corrected were sent to the participating centres for clarification.

Only cases considered by the reference pathologist to have invasive carcinoma of the breast are included. Table I shows the proportions of eligible cases and controls that were included in the analyses, and the reasons for all exclusions. The proportion of cases not interviewed was less than 10% in all centres except one, and ranged from 17.9 to 0%; the proportion of controls not interviewed was less than 10% in all but two centres, and also ranged from 17.9 to 0%. An oral contraceptive history was obtained for nearly all cases and controls. As described subsequently, variables that appeared to confound the relationship between breast cancer and oral contraceptive were controlled for, and the few cases and controls with missing values for these variables were also excluded from the analyses. Of the 2,116 cases, 75.4%, 10.9% and 5.2% had carcinomas classified as ductal, lobular and apocrine, respectively; the remaining 8.5% had one of 15 other histological types.

Unless otherwise stated, unconditional logistic regression analyses (Breslow & Day, 1980, p. 192) were utilised to estimate relative risks, adjusted for various potentially confounding variables; and all variables were entered into the regression models as categorical variables. To control for multiple factors simultaneously, a final model containing confounding variables was constructed. Variables were entered into models sequentially, one at a time, and retained if the associated $\chi^2$ test for goodness of fit was significant ($P<0.05$) and if the resultant relative risk in relation to ever use of oral contraceptives was appreciably altered. This model was then used to estimate relative risks and their 95% confidence interval under various conditions of use. Since cases tended to be older than controls, and since both the ratio of controls to cases and the prevalance of use of oral contraceptives varied among the centres, all relative risk estimates were controlled for age and centre.

**Table I** Numbers of eligible cases and controls accrued and numbers excluded from analyses by reason for exclusion

| | *Cases* | | *Controls* | |
|---|---|---|---|---|
| *Subjects* | *No.* | *%* | *No.* | *%* |
| Total accrued | 2,288 | 100.0 | 14,113 | 100.0 |
| Not interviewed | 132 | 5.8 | 767 | 5.4 |
| Oral contraceptive use unknown | 4 | 0.2 | 12 | 0.1 |
| Missing values for $\geqslant 1$ confounders | 36 | 1.6 | 262 | 1.9 |
| Included in analysis | 2,116 | 92.5 | 13,072 | 92.6 |

### Results

### *Estimates of relative risk in women who ever used oral contraceptives*

Table II shows relative risks of breast cancer, adjusted for age and centre, in relation to various previously recognised risk factors for this disease. As expected, risk of breast cancer is increased in women with a prior history of a biopsy for benign breast disease. Risk also increases with the age at which a woman first gave birth to a live child. Women who have never been pregnant and women who have previously been pregnant but have not delivered a living child are also at increased risk relative to women with a first birth at a young age. The relative risk of breast cancer decreases with increasing number of live births. Single women are at greater risk than women who have ever been married. Women whose mother or grandmother (maternal or paternal) had breast cancer are also at increased risk. Risk is reduced in women with an early menopause. Age at menarche does not appear to be an important determinant of risk. Risk is seen to increase with the number of years that a woman has spent in school. Most women in this study were also classified into one of two occupational classes to reflect socio-economic status, and women in the high occupational class were at somewhat greater risk than women in the lower class (not

**Table II** Relative risks of breast cancer in relation to various previously recognised risk factors

| Variable | Level of variable | No. of subjects Cases | Controls | Relative risk[a] (95% CI) |
|---|---|---|---|---|
| Benign breast lesion | No | 1942 | 12,666 | 1.00 |
| | Yes | 174 | 406 | 1.84 (1.51, 2.24) |
| Age at first live birth | <20 | 322 | 3,324 | 1.00 |
| | 20–24 | 817 | 4,544 | 1.55 (1.35, 1.79) |
| | 25–29 | 442 | 1,734 | 2.12 (1.80, 2.49) |
| | ⩾30 | 210 | 663 | 2.71 (2.21, 3.32) |
| | No live birth | 54 | 245 | 2.31 (1.64, 3.25) |
| | Never pregnant | 270 | 2,556 | 2.23 (1.85, 2.69) |
| | Unknown | 1 | 6 | – |
| No. of live births | None | 324 | 2,801 | 1.09 (0.94, 1.27) |
| | 1–2 | 825 | 3,809 | 1.00 |
| | 3–4 | 686 | 3,366 | 0.80 (0.71, 0.90) |
| | 5–8 | 244 | 2,513 | 0.44 (0.38, 0.52) |
| | ⩾9 | 37 | 583 | 0.29 (0.20, 0.41) |
| Marital status | Single | 198 | 2,249 | 1.00 |
| | Married | 1,655 | 9,100 | 0.80 (0.67, 0.95) |
| | Sep./div. | 167 | 1,090 | 0.79 (0.62, 1.00) |
| | Widow | 96 | 633 | 0.75 (0.57, 0.99) |
| Family history of breast cancer | No | 2,003 | 12,913 | 1.00 |
| | Yes | 113 | 159 | 3.11 (2.38, 4.06) |
| Age at menopause[b] | Pre-menop. | 1,726 | 10,591 | 1.00 |
| | ⩾50 | 62 | 288 | 0.91 (0.66, 1.26) |
| | 45–49 | 169 | 1,020 | 0.63 (0.52, 0.77) |
| | ⩽44 | 159 | 1,168 | 0.58 (0.48, 0.70) |
| | Unknown | 0 | 5 | |
| Age at menarche | ⩽11 | 160 | 836 | 1.00 |
| | 12–13 | 882 | 4,113 | 1.28 (1.05, 155) |
| | 14–15 | 728 | 4,985 | 1.10 (0.90, 1.34) |
| | ⩾16 | 341 | 3,084 | 0.98 (0.78, 1.23) |
| | Unknown | 5 | 54 | |
| Years of schooling | 0 | 157 | 1,405 | 1.00 |
| | 1–6 | 558 | 5,484 | 1.18 (0.97, 1.44) |
| | 7–12 | 932 | 4,540 | 1.66 (1.35, 2.03) |
| | ⩾13 | 469 | 1,643 | 2.49 (2.00, 3.11) |
| Socio-economic index | IV (low) | 90 | 790 | 1.00 |
| | III | 542 | 4,969 | 1.11 (0.87, 1.42) |
| | II | 609 | 4,068 | 1.33 (1.04, 1.70) |
| | I (high) | 875 | 3,245 | 2.07 (1.61, 2.65) |

[a]Adjusted for age and centre. [b]Includes both natural and artificial menopause.

shown). A four category socio-economic status index was derived from years of education and occupation (or occupation of husband if the woman was a housewife). Women of high social class according to this index were at approximately twice the risk of breast cancer compared to women in the lower social class. All of the variables in Table II, and others described below, were considered as possible confounders in the analysis of oral contraceptives in relation to risk of breast cancer.

Of the 2,116 cases, 579 (27.4%) had ever used combined oral contraceptives, and an additional 141 (6.7%) had used only oral contraceptives of an unknown type. Among the 13,072 controls, 3,671 (28.1%) had ever used combined oral contraceptives, and 756 (5.8%) others had only used unknown types. Of the women who had used combined products, 6.6% of the case users and 3.4% of the control users had also used sequential or continuous preparations; and 5.3% of the case users and 4.7% of the control users had also used oral contraceptives of unknown type. Only 3.3% of the total cases and 1.7% of the total controls had used only sequential products, and just 1.0% of the cases and 0.6% of the controls had used only continous types.

The relative risk of breast cancer in women who ever used combined oral contraceptives was estimated to be 1.15 (1.02, 1.30) after controlling for age and centre. Since most contraceptives of known type were combined preparations, it can safely be assumed that most of the unknown types were actually combined products. Also, the age and centre adjusted relative risk of breast cancer in women who ever used unknown type oral contraceptives was estimated to be 1.12 (0.93, 1.35), which is very similar to the value for combined products. Therefore, use of either combined or unknown types of oral contraceptives was assumed to represent exposure to combined oral contraceptives. Using this definition of exposure, the relative risk was again found to be 1.15, after controlling for age and centre. This definition of exposure to combined oral contraceptives was used in all subsequent analyses presented in this report.

The age adjusted estimates of the relative risks were not found to differ significantly among centres. Data from all centres were therefore combined in analyses to identify and control for confounding variables. When age, centre and the variables age at first live birth and nulliparity (as a single variable), socio-economic index, calendar year of marriage and use of an IUD were added into regression models, each was found to have confounding effects on the relative risk after controlling for the other variables in the model. These effects, however, were not in a uniform direction, and the final estimate of the relative risk based on this model was identical to that obtained when controlling only for age and centre. It is shown at the bottom of Table III.

Calendar year of marriage was considered a confounder because availability of oral contraceptives has changed over time, and there have been temporal changes in incidence rates of breast cancer in some countries. Use of an IUD was not suspected *a priori* to be a confounding variable, and controlling for all of the variables in the final model except use of an IUD resulted in an estimate of the relative risk of 1.12 (1.00, 1.26). Unless otherwise stated, the results presented subsequently are based on analyses in which use of an IUD was included as a confounding variable, but in all instances, results were similar to those obtained when estimates of relative risks were not controlled for IUD use.

A large number of other variables related to menstruation, child bearing, socio-economic status, access to medical ser-

**Table III** Relative risks of breast cancer in women who ever used combined or unknown type oral contraceptives

| Centre | Cases Users | Cases Non-users | Controls Users | Controls Non-users | Relative risk[a] (95% CI) |
|---|---|---|---|---|---|
| Australia | 37 | 27 | 436 | 241 | 0.94 (0.55, 1.60) |
| Chile | 28 | 93 | 211 | 682 | 1.03 (0.64, 1.65) |
| China | 18 | 87 | 55 | 382 | 1.28 (0.71, 2.32) |
| Colombia | 6 | 21 | 68 | 148 | 1.11 (0.42, 2.93) |
| GDR | 169 | 105 | 411 | 259 | 1.17 (0.87, 1.57) |
| Israel | 232 | 585 | 649 | 1389 | 1.05 (0.87, 1.27) |
| Kenya | 9 | 21 | 183 | 470 | 1.11 (0.50, 2.50) |
| Mexico | 26 | 59 | 315 | 748 | 1.24 (0.76, 2.02) |
| Philippines | 43 | 157 | 243 | 945 | 1.30 (0.89, 1.90) |
| Chiang Mai | 59 | 65 | 619 | 1018 | 1.70 (1.16, 2.48) |
| Chulalongkorn | 40 | 69 | 608 | 871 | 0.87 (0.58, 1.31) |
| Siriraj | 53 | 107 | 629 | 1492 | 1.30 (0.91, 1.84) |
| Total | 720[c] | 1396 | 4427[d] | 8645 | 1.15 (1.02, 1.29)[b] |

[a]Controlled for age, age at first live birth, socio-economic index, year of marriage, and use of an IUD. [b]Controlled also for centre. [c]Including 141 users of unknown type. [d]Including 756 users of unknown type.

vices and exposure to exogenous hormones were also considered as possible confounders, and none had an additional influence on the relative risk in users of oral contraceptives. These additional variables include: numbers of pregnancies and live births; ages at first breast feeding and last pregnancy; months of lactation; marital status; outcome of first pregnancy; years of schooling; occupation; ethnic origin (Israel only); husband's years of schooling; husband's occupation; method of payment for medical care; calendar years of birth and menarche; ages at menarche and menopause; history of benign breast disease; family history of breast cancer; history of infertility; source of referral to hospital; calendar year of interview; use of reserpine; numbers of chest X-rays and prior pap smears; several indices of use of hormones for non-contraceptive purposes; use of sequential, continuous, post-coital or injectable steroid contraceptives; duration of residence at current address; and consumption of alcoholic beverages.

Since the value of 1.15 for the relative risk, although not large, was of borderline statistical significance, and hence somewhat at variance with results from most other studies, possible reasons for this finding were investigated. An interaction term for centre and use of oral contraceptives was included in the final model to provide separate estimates of the relative risk for each individual centre. These are shown in Table III. Although these estimates did not vary significantly, that for Chiang Mai was larger than those for the other centres and was the only one with a lower 95% confidence limit great than 1. The data from Chiang Mai were therefore further evaluated for evidence of possible bias. The observed increase in relative risk could not be attributed to confounding by any additional variables not included in the analyses shown in Table III, variation in data collected by different interviewers, or under-representation of oral contraceptive use in controls in any particular diagnostic category.

The determinants of oral contraceptive use may vary among countries and cultures. The variables identified as confounders from analyses of data from all centres combined may therefore not be the most important confounders in some individual centres. Separate analyses of the data from each centre were therefore performed, using the same stepwise regression technique that was utilised to analyse the data from all centres combined. The values for the resultant relative risks did not vary appreciably from those in Table III. A $\chi^2$ test for heterogeneity among the individual estimates for each centre was not significant. Based on an average of the individual estimates for each centre, weighted by the inverse of their variance (Prentice & Thomas, 1987), a summary relative risk of 1.13 (1.00, 1.28) was obtained. Since this estimate is very close to that of 1.15 obtained from the combined analysis of data from all centres, and for ease in computation, the model developed for all centres combined was used for most subsequent analyses.

*Duration, latency and recency of use*

Table IV shows estimates of the relative risk of breast cancer in relation to years of use (duration), time since first use (latency) and time since most recent exposure (recency). A statistically significant trend of increasing risk with years of use was observed. Among users, risk declined with years since first use, but the trend was not significant. The relative risk is highest in current users, and among users a steady and significant decline in risk with time since last exposure is evident.

If a woman gave a history of having used oral contraceptives, an attempt was made to obtain additional or confirmatory information from her medical records on brand names and periods of use. This procedure would not alter the classification of a woman as having ever used oral contraceptives, because medical records of women without a history of use were not reviewed, and if use by a woman who claimed to have taken oral contraceptives could not be confirmed from medical records, she was still considered to be a user.

**Table IV** Relative risks of breast cancer in relation to duration of use, time since first use, and time since last use of combined oral contraceptives[a]

| *Category of use* | *Cases* | *Controls* | *Relative risk*[b] *(95% CI)* |
|---|---|---|---|
| Non-users | 1395 | 8640 | 1.00 |
| Years of use[c] | | | |
| <1 | 247 | 1670 | 1.12 (0.95, 1.31) |
| 1–2 | 194 | 1397 | 1.01 (0.82, 1.23) |
| 3–8 | 126 | 735 | 1.17 (0.97, 1.42) |
| >8 | 123 | 417 | 1.56 (1.23, 1.98) |
| *P* value for trend test | | | 0.007 |
| Years since first use[d] | | | |
| <3 | 44 | 494 | 1.39 (0.98, 1.96) |
| 3–9 | 194 | 1293 | 1.30 (1.08, 1.57) |
| 10–15 | 300 | 1569 | 1.14 (0.97, 1.33) |
| >15 | 162 | 930 | 1.00 (0.82, 1.21) |
| *P* value for trend test[g] | | | 0.12 |
| Months since last use[e] | | | |
| Current users[f] | 127 | 747 | 1.66 (1.32, 2.08) |
| 4–35 | 120 | 751 | 1.41 (1.13, 1.77) |
| 36–108 | 234 | 1374 | 1.16 (0.98, 1.37) |
| ⩾109 | 213 | 1388 | 0.91 (0.77, 1.08) |
| *P* value for trend test[g] | | | 0.0001 |

[a]Users of unknown type oral contraceptives assumed to have used combined type. [b]Adjusted for age, centre, age at first live birth, socio-economic index, year of marriage, and use of an IUD. One case and six controls with unknown age at first live birth excluded. [c]Excluding 30 cases and 207 controls with unknown years of use. [d]Excluding 20 cases and 140 controls with unknown years since first use. [e]Excluding 26 cases and 166 controls with unknown years since last use. [f]Including women who discontinued use in the past three months. [g]Trend test based on data from exposed subjects.

These procedures would also have had a minimal effect on classifying a woman as ever having used combined oral contraceptives (including unknown types), since few other types of oral contraceptives were available, and few women erroneously reporting use of combined preparations would have been reclassified as having used a non-combined product, or vice versa. Therefore, the estimated values of the relative risk of breast cancer in women who ever used combined oral contraceptives could not have been appreciably influenced by any differences in the proportion of cases or controls whose oral contraceptive histories were supplemented by information from medical records. Such differences could, however, alter estimated values of the relative risk in relation to such features of use as duration, latency or recency. Information from medical records was obtained for 27% of the case users and 18% of the control users. These percentages varied widely among participating centres, from 0 to 94% of the cases and 0 to 89% of the controls. Information was most frequently obtained from the medical records of long-term and current or recent users in both the case and control groups. However, results similar to those in Table IV were obtained separately from countries in which information from medical records was obtained for relatively high and low proportions of users, and in individuals whose use was ascertained solely from interviews and from both interviews and medical records. Any differences between cases and controls in the fidelity of the efforts to obtain information on use of oral contraceptives from medical records is thus not a likely explanation for any increased risks that are observed.

Table V shows that the possible increase in risk in long-term users of oral contraceptives is evident in all categories of latency (years since first use). However, Table VI shows that this possible increased risk in long-term users is due to an association between years of use and months since last use (recency). No increase in risk with duration of use is seen in any category of recency, but risk declines with time since last use for all categories of duration.

*Use in women with risk factors for breast cancer*

Exploratory analyses using the model developed for all women combined resulted in relative risk estimates in rela-

**Table V** Relative risks[a] of breast cancer in relation to duration of use and years since first use of combined oral contraceptives[b]

| | *Years of use* | | | |
|---|---|---|---|---|
| *Years since first use* | <1 | 1–2 | 3–8 | >8 |
| <3 | 1.41 | 1.35 | – | – |
| 3–9 | 1.28 | 1.36 | 1.22 | 2.48 |
| 10–15 | 0.97 | 0.93 | 1.33 | 1.68 |
| >15 | 1.05 | 0.90 | 0.68 | 1.35 |

[a]Risks are relative to risk in non-users, adjusted for age, centre, age at first live birth, socio-economic index, year of marriage and use of an IUD. [b]Users of unknown type oral contraceptives are assumed to have used combined type.

**Table VI** Relative risks[a] of breast cancer in relation to duration of use and years since last exposure to combined oral contraceptives[b]

| | *Years of use* | | | |
|---|---|---|---|---|
| *Months since last use* | <1 | 1–2 | 3–8 | >8 |
| Current users[c] | 2.07 | 1.41 | 1.19 | 2.16 |
| 4–36 | 1.23 | 1.63 | 1.53 | 1.38 |
| 37–108 | 1.23 | 1.10 | 1.20 | 1.12 |
| ⩾109 | 1.01 | 0.88 | 0.60 | 0.76 |

[a]Risks are relative to risk in non-users, adjusted for age, centre, age at first live birth, socio-economic index, year of marriage and use of an IUD. [b]Users of unknown type oral contraceptives are assumed to have used combined type. [c]Includes women who discontinued use in the past 3 months.

tion to use of oral contraceptives that were higher in women under than over age 35. Some risk factors for breast cancer may have effects in young women opposite to those in older women (Janerich & Hoff, 1982); and in this study the variables most strongly associated with breast cancer were different for women under and over age 35 (not shown). A separate model was therefore developed to estimate relative risks of breast cancer in relation to combined oral contraceptives in women under age 35. This was developed in the same manner as the model for women of all ages considered. The final model for women less than 35 contained the following variables: age, centre, husband's occupation, years of schooling, number of live births, age at first live birth, year of birth and family history of breast cancer. As shown in the upper portion of Table VII, the estimated relative risk of breast cancer in women under age 35 who ever used oral contraceptives is 1.26. The estimated relative risk for older women, using the original model to analyse data separately from such women, was 1.12. These two values do not differ significantly ($P$ value of $\chi^2$ test for heterogeneity = 0.38). The mean of these two estimates weighted by the inverse of their variance provides an overall estimate of 1.14 (1.02, 1.27). Because this estimate is similar to the overall estimated relative risk of 1.15, the original model was used in analyses that included women of all ages. Analyses of data on women under age 35 were based on the model developed for that age group.

A relative risk of greater than 1 in women under age 35 was observed in seven of the 10 centres from which sufficient data were available to provide an estimate of the relative risk in such women, and a $\chi^2$ test for heterogeneity showed the variation in the estimates for the various centres to be explainable on the basis of chance ($P = 0.36$). The relative risks in relation to years of use, time since first use and time since last use were not consistently greater in women less than 35 than in older women, and the trend of increasing risk with duration of use shown in Table IV for women of all ages was no stronger in the younger than older groups of women.

As shown in Table VII, no statistically significant interactions were observed between use of oral contraceptives and age at first live birth and nulliparity, outcome of first pregnancy, socio-economic index or family history of breast cancer.

The effect on risk of breast cancer of use of oral contraceptives before and after a biopsy for a benign breast lesion was assessed from data on the 174 cases and 406 controls who gave a history of such a lesion. Use before their benign lesion was reported by 31 cases and 77 controls; and use afterwards was reported by 26 cases and 68 controls. The relative risk of breast cancer was not significantly enhanced either in women who used oral contraceptives before their prior breast biopsy (RR = 1.30; 95% CI = 0.75, 2.27) or after (RR = 0.97; 95% CI = 0.54, 1.72). These estimates were calculated controlling for the same variables as in Table III.

**Table VII** Relative risks of breast cancer in relation to use of combined oral contraceptives[a] in women characterised by various risk factors for breast cancer

| | *Cases* | | *Controls* | | | *P value of $\chi^2$ test for* |
|---|---|---|---|---|---|---|
| *Variable* | *User* | *Non-user* | *User* | *Non-user* | *Relative risk[b] (95% CI)* | *heterogeneity* |
| Age | | | | | | |
| <35 | 160 | 141 | 1613 | 2722 | 1.26 (0.95, 1.66) | 0.38 |
| ⩾35 | 560 | 1255 | 2814 | 5923 | 1.12 (0.98, 1.27) | |
| Age at first live birth[c] | | | | | | |
| <24 | 424 | 715 | 3068 | 4800 | 1.17 (1.02, 1.36) | 0.36 |
| 25–29 | 171 | 271 | 670 | 1064 | 1.27 (1.01, 1.61) | |
| >29 | 69 | 141 | 234 | 429 | 1.13 (0.79, 1.60) | |
| No live birth | 19 | 35 | 108 | 137 | 0.75 (0.38, 1.47) | |
| Never pregnant | 37 | 233 | 346 | 2210 | 0.84 (0.56, 1.25) | |
| Outcome of first pregnancy | | | | | | |
| Viable | 577 | 979 | 3553 | 5718 | 1.22 (1.08, 1.39) | 0.34 |
| Non-viable | 87 | 149 | 420 | 580 | 0.96 (0.70, 1.32) | |
| Nulliparous | 56 | 268 | 454 | 2347 | 0.80 (0.56, 1.13) | |
| Socio-economic index | | | | | | |
| IV (low) | 19 | 71 | 156 | 634 | 1.86 (1.07, 3.24) | 0.33 |
| III | 142 | 400 | 1498 | 3471 | 1.13 (0.91, 1.40) | |
| II | 202 | 407 | 1420 | 2648 | 1.14 (0.94, 1.39) | |
| I (high) | 357 | 518 | 1353 | 1892 | 1.12 (0.94, 1.32) | |
| Family history of breast cancer | | | | | | |
| No | 669 | 1334 | 4349 | 8564 | 1.13 (1.01, 1.27) | 0.65 |
| Yes | 51 | 62 | 78 | 81 | 1.27 (0.75, 2.16) | |

[a]Users of unknown type oral contraceptives are assumed to have used combined types. [b]Risks are relative to risk in non-users. All are adjusted for age, centre, age at first live birth, socio-economic index, use of an IUD and year of marriage, except for the relative risk in women less than 35 years old, which is adjusted for age, centre, husband's occupation, years of schooling, number of live births, age at first live birth, year of birth and family history of breast cancer. [c]Excluding one case and six controls with unknown age at first live birth.

*Use in developed and developing countries*

Since one purpose of this study was to determine whether results of studies of oral contraceptives and breast cancer conducted in developed countries are similar to results from developing countries, it was decided *a priori* to estimate relative risks separately for women in developed and developing nations. For this purpose data from the three most economically developed countries in this study were combined, as were data from the remaining seven less developed countries. The three countries that were considered developed for this purpose were Australia and Israel, which have high rates of breast cancer, and the German Democratic Republic, which has moderately high rates (Muir *et al.*, 1987). The seven other countries are either known to have lower incidence rates of breast cancer, or can reasonably be assumed to have low rates because of their adjacency and economic similarity to known low incidence nations (Waterhouse *et al.*, 1982; Muir *et al.*, 1987).

As shown in Table VIII, the relative risk in women who ever used oral contraceptives is somewhat greater for women in developing than developed countries, although the observed difference could have occurred by chance ($P = 0.22$). The values of the relative risks in most categories of duration, latency and recency are also higher in the developing countries, although the differences are small, and also not statistically significant. The trends of increasing risk with duration of use and times since first and last exposure are also somewhat stronger in the developing countries.

No trends of increasing or decreasing relative risk in oral contraceptive users with age were observed in either group of countries. Relative risks in all women under age 35 were similar in developing and developed countries, although a relative risk of 3.05 (1.69, 5.51) was found in the youngest group of women (less than 29) in developing countries (based on 23 exposed cases and 644 exposed controls).

*Use in early reproductive life*

The observed effect of oral contraceptives on risk of breast cancer did not vary significantly among women who first used these products at various ages. The highest relative risk (1.42; 95% CI = 1.04, 1.58) was observed in women who first began using them after the age of 35.

Relative risks of breast cancer in relation to duration of use of oral contraceptives before age 25 are shown in the upper portion of Table IX. A steady trend of increasing risk with years of use is not evident ($P$ value of test for trend = 0.31), but the relative risks for users of more than 2 years' duration are increased and of borderline statistical significance. Most exposure to oral contraceptives before age 25 occurred in women under age 35. In such women, risk also did not steadily increase with months of use before age 25, although the relative risk for women with more than 3 years of use was 1.63 (0.95, 2.80), based on 26 exposed cases and 189 exposed controls. Results regarding use before age 25 were similar in developed and developing countries.

As shown in the lower portion of Table IX, no trend of increasing risk with duration of use of oral contraceptives before a woman's first live birth was observed, although a small relative risk of 1.24, with confidence limits that include 1.0, was found in women with such use for over 2 years. Because use before a woman's first birth has been associated with an increased risk of breast cancer in several studies, the small elevation in relative risk estimated in this investigation was evaluated further. The possible increase in risk was found to be confined to women who had had a non-viable pregnacy before their first live birth. In these women, risk increased with duration of use before their first live birth, and in women with such use for over 2 years, a relative risk of 2.56 (1.05, 6.23) was estimated, based on 10 exposed cases and 29 exposed controls. There were too few women with sufficient use before their first live birth to determine whether risk is enhanced in long-term users after a prolonged time since initial exposure. Only 2 cases and 11 controls had initially been exposed to oral contraceptives over 10 years previously, and had used them for more than 5 years before their first live birth.

*Tumour size and stage*

It has been suggested (Skegg, 1988) that women who use oral contraceptives may be more likely to have their breast cancer detected at an early stage than non-users. If so, then this would result in an observed excess relative risk of small, early stage tumours, and this might be particularly evident in younger age women if there was a shortening of time from onset of disease to detection. If this source of bias were

**Table VIII** Relative risks of breast cancer in developed and developing countries in relation to various measures of use of combined oral contraceptives

| | *Developed countries*[a] | | | *Developing countries*[b] | | |
|---|---|---|---|---|---|---|
| | *No. of subjects* | | *Relative risk*[c] | *No. of subjects* | | *Relative risk*[c] |
| *Category of use* | *Cases* | *Controls* | *(95% CI)* | *Cases* | *Controls* | *(95% CI)* |
| Non-user | 716 | 1888 | 1.00 | 679 | 6752 | 1.00 |
| Any use | 438 | 1496 | 1.07 (0.91, 1.26) | 282 | 2930 | 1.24 (1.05, 1.47) |
| Years of use[d] | | | | | | |
| <1 | 134 | 418 | 1.07 (0.85, 1.35) | 113 | 1252 | 1.17 (0.93, 1.46) |
| 1–3 | 101 | 439 | 0.88 (0.68, 1.13) | 93 | 958 | 1.30 (1.01, 1.66) |
| 4–8 | 88 | 307 | 1.11 (0.84, 1.47) | 38 | 428 | 1.09 (0.76, 1.56) |
| >8 | 89 | 206 | 1.39 (1.03, 1.88) | 34 | 211 | 1.88 (1.27, 2.78) |
| *P* value for trend test | | | 0.13 | | | 0.01 |
| Years since first use[e] | | | | | | |
| <3 | 18 | 101 | 1.25 (0.71, 2.19) | 26 | 393 | 1.46 (0.94, 2.27) |
| 3–9 | 105 | 400 | 1.08 (0.83, 1.40) | 89 | 893 | 1.56 (1.21, 2.03) |
| 10–15 | 200 | 560 | 1.17 (0.95, 1.45) | 100 | 1009 | 1.12 (0.88, 1.43) |
| >15 | 96 | 350 | 0.89 (0.68, 1.16) | 66 | 580 | 1.13 (0.85, 1.50) |
| *P* value for trend test[g] | | | 0.44 | | | 0.10 |
| Months since last use[f] | | | | | | |
| <3 and current | 78 | 1888 | 1.53 (1.12, 2.10) | 49 | 492 | 1.86 (1.34, 2.60) |
| 4–35 | 76 | 262 | 1.31 (0.96. 1.77) | 44 | 489 | 1.55 (1.10, 2.18) |
| 36–108 | 143 | 457 | 1.07 (0.85, 1.35) | 91 | 917 | 1.28 (1.00, 1.64) |
| ⩾109 | 117 | 409 | 0.85 (0.67, 1.07) | 96 | 979 | 1.00 (0.79, 1.28) |
| *P* value for trend test[g] | | | 0.01 | | | 0.002 |

[a]Includes Australia, German Democratic Republic and Israel. [b]Includes Chile, Colombia, China, Kenya, Mexico, Philippines and Thailand. [c]Controlled for age, centre, age at first live birth, socio-economic index, year of marriage and use of an IUD. One case and six controls with unknown age at first live birth excluded. [d]Excluding 30 cases and 207 controls with unknown duration of use. [e]Excluding 20 cases and 140 controls with unknown time since first use. [f]Excluding 26 cases and 166 controls with unknown time since last use. [g]Trend test based only on data from exposed subjects.

**Table IX** Relative risks of breast cancer in relation to duration of use of oral contraceptives before age 25 years and before first live birth

| *Time of use* | *Months of use* | *No. of subjects* Cases | Controls | *Relative risk*[a] *(95% CI)* |
|---|---|---|---|---|
| Before age 25 years[c] | None | 1,908 | 11,104 | 1.00 |
| | <12 | 82 | 881 | 1.02 (0.79, 1.32) |
| | 12–24 | 37 | 421 | 0.79 (0.55, 1.14) |
| | 25–36 | 29 | 207 | 1.51 (0.98, 2.31) |
| | ⩾37 | 29 | 243 | 1.49 (0.97, 2.28) |
| Before first live birth[d] | None | 1,691 | 9,660 | 1.00 |
| | Any | 87[e] | 548[e] | 0.91 (0.69, 1.20)[b] |
| | <2 | 56[f] | 405[f] | 0.82 (0.59, 1.13)[b] |
| | ⩾2 | 29 | 110 | 1.24 (0.78, 1.97)[b] |

[a]Controlled for age, centre, age at first live birth and nulliparity, socio-economic index, year of marriage and use of an IUD. [b]Controlled also for use of oral contraceptives after a first live birth. [c]Excludes 31 cases and 216 controls with unknown use before age 25. [d]Excludes 325 cases and 2,825 controls with no live birth. [e]Excludes 13 cases and 39 controls with unknown use before first life birth. [f]Excludes two additional cases and 33 additional controls with unknown duration of use before first live birth.

operating one would also expect to observe an increase in relative risk primarily in relation to current and recent users, and such users would also tend to have small, early stage tumours.

Among all cases in this study, 43.3% of those who had ever used oral contraceptives presented with small tumours (<3 cm), compared to 38.3% of non-users. However, after adjusting for confounding variables considered in previous analyses, relative risks were not found to be enhanced predominantly for smaller tumours in all women who ever used oral contraceptives, in users under age 35, or in users in developing countries. Risk in users was also not preferentially enhanced for tumours confined to the breast compared to tumours with local spread or distant metastasis. Also, the increases in risk in relation to long-term, current and recent use of oral contraceptives were observed for tumours of all sizes. It is therefore unlikely that selective detection of cases in women who used oral contraceptives can explain the observed overall increase in risk, or the possible increases in young women, in developing countries, or in long-term, recent and current users.

Tumours in women who used oral contraceptives for more than 36 months before they were 25 years old tended to be small (73.9% of 23 of known size were less than 3 cm in diameter), although tumours in users of 25–36 months' duration, who had an equally high relative risk, did not (37.5% of 24 tumours were less than 3 cm). Thus, preferential screening in young women who have been long-term users of oral contraceptives may partly account for the findings in the upper part of Table IX, but it is probably not the total explanation. On the other hand, women with a prior non-viable pregnancy before their first live birth, who used oral contraceptives before their first live birth, more frequently had small tumours (less than 3 cm in diameter) than similar women who had not used oral contraceptives before their first live birth (59.2% of 13 tumours of known size *vs* 33% of 15 tumours). Also, in users before their first live birth with a prior non-viable pregnancy, age and centre adjusted relative risks were 4.44 (1.35, 14.6), 1.33 (0.26, 6.95) and 0.74 (0.08, 7.05) for tumours that were <3, 3–4 and >4 cm in diameter, based respectively on nine, three and one exposed cases, and 47 exposed controls. The small increase in risk in relation to use before a first live birth (Table IX) may therefore be due to selective early diagnosis of breast cancer in women with such use, if they had had a prior non-viable pregnancy.

### *Composition of oral contraceptives*

Details of results in relation to oral contraceptives of varying compositions will be the subject of a separate report, and only observations of value in interpreting the findings presented in this paper are summarised here. Relative risks of breast cancer in relation to 22 different combined oral contraceptives were estimated from data on non-users and women who had used a single (known) combined product. Relative risks in women who ever used these products were less than 1 for nine formulations, and greater than 1 for 13. The relative risks ranged from 0.74 to 1.43 among 21 pill types, and a relative risk of 8.19 (1.94, 34.6), based on four exposed cases and five exposed controls, was observed for one product (1 mg lynestrenol plus 0.1 mg mestranol). The products associated with the higher relative risks tended to be those used more frequently both in women under age 35 and in developing countries. However, the products associated with high relative risks did not differ consistently from those associated with lower relative risks by type of oestrogen or progestagen, or by the dosages of either of these constituents. Also, it was possible to estimate relative risks in relation to three specific formulations both for women under age 35 and for older women; and the relative risks in users of all three products were higher in the younger than in the older age group. Similarly, the relative risks were higher in developing than developed countries for both of the formulations for which it was possible to make individual estimates of the relative risk in each of the two groups of countries. It is therefore unlikely that any differences in relative risks associated with oral contraceptives in developed and developing risk countries and in younger and older women are due to use of different oral contraceptives with different degrees of carcinogenicity for the breast.

There were too few data for analysis to determine whether the enhanced risks shown in Table IX are specific for particular types of oral contraceptives.

### Discussion

Estimates of the relative risk of breast cancer in women who ever used oral contraceptives were recently estimated to be 1.0 (0.9, 1.1) and 1.0 (0.8, 1.1) based on combined data from 16 previous case–control and four cohort studies, respectively (Thomas, 1988). Nearly all of these prior studies were conducted in developed countries with relatively high rates of breast cancer. Although the relative risk of 1.15 (1.02, 1.29) estimated in this study is not incompatible with these summary estimates, it is higher than that found in most prior investigations. The estimates for women in developed and developing countries in this study did not differ significantly, but the value for developing countries was the higher of the two, and contributed to most of the possible small overall elevation in risk that was observed. The estimated relative risk of 1.07 (0.81, 1.26) in women in the three developed countries who ever used oral contraceptives is close to the summary estimates of Thomas (1988). Also, the relative risk of 0.9 in women in these three countries who were initially exposed over 15 years previously is identical to the value estimated from the eight prior case–control studies that provided relative risks for women initially exposed from over 10 to over 20 years in the past (Thomas, 1988). In contrast, the point estimate of the relative risk for women in the developing countries was 1.24 (1.05, 1.47) in women who ever used oral contraceptives and this is greater than values obtained from all but four (Kelsey *et al.*, 1978; Pike *et al.*, 1981; Miller *et al.*, 1989; UK National Case–Control Study Group, 1989) of 19 previous case–control studies, and from all but one (Kay & Hannaford, 1988) of four previous cohort studies (Thomas, 1988; Kay & Hannaford, 1988; Vessey *et al.*, 1989) conducted in developed countries. Results from the few studies of oral contraceptives and breast cancer in developing countries (Lee *et al.*, 1987; Yuan *et al.*, 1988) have yielded inconsistent results. Since the results from this study, particularly those from developing countries, may be at variance with results from most prior investigations, possible reasons for a spurious increase in risk must be considered.

Because this study was conducted in part in countries with limited medical care facilities, some cases may not come to medical attention. If such cases were also those less likely to have received other medical services, including family planning services, then the (hospitalised) cases included in this study would be more likely to have used oral contraceptives than the hypothesised missed cases, and this could result in a spurious increase in relative risk in relation to oral contraceptive use. This possible source of bias was anticipated when the study was planned, and resulted in the decision to select hospital controls, rather than controls from the same village or neighbourhood as the cases. This as least partly controlled for (unknown) factors that determine entry into the medical care system and admission to the hospitals in which the study was conducted. Information was also obtained on various indices of medical care utilisation, such as prior pap smears and chest X-rays, and control for these factors did not alter the estimated values of the relative risks in oral contraceptive users.

Conversely, a bias in the opposite direction could have resulted because some hospitals served both as referral centres for such serious and relatively unusual conditions as breast cancer, and also as local, general hospitals; and if access to contraceptive services is better in areas in proximity to the hospital than in more distant regions, and if cases came from a wider catchment area than the controls, then this would lead to a spuriously low relative risk. To reduce the possibility of this source of bias, cases and controls were restricted to defined geographical areas served by the hospitals. These areas were often quite large, however. To reduce further the possibility of this source of bias, a detailed residential history was ascertained for all study subjects, so that estimates of relative risks could be controlled for place of residence (urban centre, town, rural village) and mobility. Controlling for such variables did not alter the results of this study.

Another possible explanation for a spurious increase in relative risk is that the hospital controls had diseases that were associated with under-use of oral contraceptives. To reduce this source of bias, individuals who were hospitalised for conditions known or perceived to be related to use of oral contraceptives, or contra-indications to their use, were not eligible for selection as controls. Furthermore, the controls consisted of women with a large variety of medical conditions, and use of oral contraceptives did not vary greatly among women in the various diagnostic categories. Under-utilisation of oral contraceptives in some diagnostic groups of controls therefore cannot explain the observed increase in relative risk.

Bias due to more intensive screening for breast cancer in oral contraceptive users than in non-users also cannot explain the overall increase in relative risk observed, or the increase in long-term users, or in recent and current users. The relative risks in relation to these features of use did not vary appreciably by tumour size or stage of disease at diagnosis. Also, non-invasive carcinomas were not considered in this report, in part because they would be the most likely malignancies to be detected by screening.

Another possible reason for a spuriously enhanced relative risk is more complete recall of prior use of oral contraceptives by cases than controls. Utilisation of hospitalised women as controls reduced the possibility of this occurring. Furthermore, if this had occurred, one would also expect to observe an enhanced relative risk of other cancers in relation to oral contraceptives. On the contrary, use of oral contraceptives has been found in this study to be associated with a reduced risk of cancer of the endometrium (WHO Collaborative Study of Neoplasia and Steroid Contraceptives, 1988) and ovary (WHO Collaborative Study of Neoplasia and Steroid Contraceptives, 1989) and the estimates of the relative risks of these neoplasms in users of oral contraceptives are similar in magnitude to those from prior studies (Prentice & Thomas, 1987). Also, results from this study in relation to such details of use as duration, recency and latency were similar in individuals whose oral contraceptive use was ascertained only from interviews and in those whose information on use was supplemented by a review of medical records.

Spurious results could also have resulted from confounding by risk factors for breast cancer that are also related to use of oral contraceptives. This is unlikely because most of the known risk factors for breast cancer were considered, and information on them was most probably correctly ascertained, because these factors were found to be related to breast cancer in this study (Table II). Possible risk factors that were not considered are obesity and high fat diet. Obesity is an unlikely confounder in this study because most women were relatively young, and obesity has been most strongly and consistently related to breast cancer in postmenopausal women. Although national rates of breast cancer have been correlated with consumption of animal fats and other meat products, a high fat diet has been only weakly and inconsistently related to breast cancer in case–control studies, perhaps because the variation in fat intake within countries is relatively small in comparison with international variations in fat consumption (Prentice *et al.*, 1990). Controlling for centre in this study effectively controlled for differences in fat intake among centres. Control for various indices of socio-economic status would have partly controlled for differences in fat intake between cases and controls within centres. It is thus unlikely that the variation in fat intake among individuals within centres, and the strength of the association between fat intake and breast cancer, would be of sufficient magnitude that residual confounding by fat intake could explain the increased relative risk in users of oral contraceptives.

Chance variation is also unlikely to be the sole explanation for our findings. The 95% confidence intervals of the relative risks in women who ever used oral contraceptives do not include unity, when based either on data from all subjects in the study, or on data from residents of the developing countries; the relative risks were greater than 1 in 10 of the 12 centres, including eight of the nine in developing populations.

Although a combination of chance, and minor sources of bias and confounding, could account for our results, a causal interpretation must also be considered. Although risk was observed to be highest in the longest use category (Table IV), an increase with duration of exposure was not observed after stratifying on months since most recent exposure (Table VI). This absence of a trend of increasing risk with duration of use, and lack of an increase in risk with time since first use, are not observations that one would expect if oral contraceptives were involved in initiating a carcinogenic process. In this study, an enhanced risk was observed in current and recent users, and there was a decline in risk with time since last use. Similar observations have been reported at least to some extent from four previous investigations (Fasel *et al.*, 1975; Brinton *et al.*, 1982; Harris *et al.*, 1982; Meirik *et al.*, 1986), but not from two others (Vessey *et al.*, 1983; Cancer and Steroid Hormone Study, 1986). Such a relationship would be compatible with a late stage carcinogenic effect of oral contraceptives on the breast. If such an effect exists, it is not potentiated by other known risk factors for breast cancer, because the relative risk in relation to oral contraceptives was not greater in individuals with than without such risk factors as older age, older age at first birth, nulliparity, high socio-economic status, family history of breast cancer (Table VII) or history of benign breast disease. Prior studies have also not consistently demonstrated interactions between oral contraceptives and age at first birth, parity, family history of breast cancer or prior benign breast disease (Thomas, 1988). There is thus little or no reason to advise women to avoid using oral contraceptives if they have one or more of these features that are associated with an increased risk.

The difference in the relative risks of 1.07 and 1.24 observed in developed and developing countries, respectively, could be due to chance or to unrecognised sources of bias or confounding that were operative primarily in the less developed countries in which this study was conducted. Alternatively, these findings are compatible with oral contraceptives exerting a small additive effect on risk of equal magnitude in countries with varying underlying rates of breast cancer.

Thus, even if the relative risks are truly higher in the developing countries, the numbers of cases per 100,000 women years of exposure attributable to use of oral contraceptives would not be greater than in the developed countries because of their lower underlying incidence rates.

If oral contraceptives were to enhance risk of breast cancer by only a small absolute number of additional cases per 100,000 women years of use, then it is not surprising that prior studies conducted largely in high rate countries have failed to detect such small increases in risk. Epidemiological methods currently available may not be sufficiently sensitive to do so. Conducting studies in low risk populations is one way to enhance the likelihood of detecting a true, but small, absolute increase in risk, because the relative risk in such populations would be larger than in higher risk populations. The results of this study serve to demonstrate the utility of this approach. They also indicate that further studies of breast cancer and oral contraceptives in low risk populations are warranted to determine whether the findings presented in this report can be replicated.

The possibility that use of oral contraceptives at an early age, or before the birth of a woman's first child, enchances risk of breast cancer has been the subject of considerable study and debate in recent years. Prior investigations have yielded inconsistent results. An increased risk in women who used oral contraceptives before age 25 was found in three studies (Pike *et al.*, 1983; Olsson *et al.*, 1985; Meirik *et al.*, 1986), but not in four others (Paul *et al.*, 1986; Cancer and Steroid Hormone Study, 1986; Miller *et al.*, 1986, 1989). The observed increase in risk in this study, of borderline statistical significance, in women who had used oral contraceptives before age 25 for over 2 years is somewhat supportive of the notion that such use can enhance the development of breast cancer; but the absence of a significant trend of increasing risk with duration of use is not. By considering the size of the tumours of diagnosis, evidence was provided that the observed enhanced risk in long-term users before age 25 is probably not due solely to preferential screening for breast cancer in such women.

Significantly elevated relative risks in women who used oral contraceptives before the birth of their first child have been reported from four independent investigations (Paffenbarger *et al.*, 1980; Pike *et al.*, 1981; Harris *et al.*, 1982; McPherson *et al.*, 1987), but not from eight others (Vessey *et al.*, 1982; Hennekens *et al.*, 1984; Stadel *et al.*, 1985; Lipnick *et al.*, 1986; Meirik *et al.*, 1986; Paul *et al.*, 1986; Miller *et al.*, 1986; Jick *et al.*, 1989). A small increase in risk in women who used oral contraceptives for more than 2 years before their first live birth was observed in this study, but this increase was found only in women whose live birth was preceded by a pregnancy with a non-viable outcome, and evidence has been presented that suggests that this observation may be due to preferential screening of such users for breast cancer. No satisfactory explanation has yet been found for the inconsistent results among previous studies regarding use before a woman's first birth. The findings from this study offer a possible explanation, and it would be useful if others would attempt to replicate them. Results from two recent case–control studies (Miller *et al.*, 1989; UK National Case–Control Study Group, 1989) showed increased relative risks of breast cancer in young women in relation to use of oral contraceptives, irrespective of whether the use was before or after the birth of the woman's first child. The findings from this study are broadly consistent with these results.

The data collection centres, and the principal investigator (PI), co-investigator (CI) and pathologist (P) at each participating centre in alphabetical order by country, are as follows:

*University of Sydney*, Department of Public Health, Sydney, Australia: Geoffrey Berry (PI), Robert MacLennan (CI), Rodney Shearman (CI), Tatiana Jelihovsky (P), Joan Cooper Booth (P).
*University of Chile*, Faculty of Medicine, Hospital Jose Joaquin Aguirre, Department of Obstetrics and Gynaecology, and the Ministry of Health, Hospital Salvador, Department of Obstetrics and Gynaecology, Santiago, Chile: Ramiro Molina (PI), Luis Martinez (CI), Oriana Salas (CI), Alfredo Dabancens (P).
*Shanghai Institute of Planned Parenthood Research*, Shanghai, China: Chen Zhiheng (PI), Tao Yun (CI), Hu Yong Wei (P).
*Hospital Universitario*, WHO Collaborative Centre for Research in Human Reproduction, Cali, Colombia: Alvaro Cuadros (PI), Nubia Aristizabal (P).
*Central Institute of Cancer Research, Academy of Sciences of the German Democratic Republic*, Berlin, GDR: K. Ebeling (PI), P. Nishan (CI), D. Kunde (P).
*Chiam Sheba Medical Centre*, Department of Clinical Epidemiology, Tel Hashomer, Israel: Baruch Modan (PI), Elaine Ron (CI), Ester Alfandary (CI).
*University of Nairobi*, Nairobi Centre for Research in Reproduction, Nairobi, Kenya: J.G. Mati (PI), Patrick Kenya (CI), Alfred Kungu (P), D. Gatei (P).
*Hospital General de Mexico*, Mexico City: Hector Rodriguez Cuevas (PI), Socorro Benavides Salazar (CI), Antonio Palet (P), Patricia Ontiveros (P).
*University of the Philippines*, College of Medicine, Manila, Philippines: Ruben A. Apelo (PI), Julietta R. de la Cruz (CI), Jose Baens (CI), Benita Javier (P).
*Chiang Mai University*, Faculty of Medicine, Chiang Mai, Thailand: Suporn Silpisornkosol (PI), Tieng Pardthaisong (CI), Nimit Martin (CI), Choti Theetranont (P).
*Chulalongkorn University*, Faculty of Medicine, Department of Obstetrics and Gynaecology, WHO Collaborating Centre for Research in Human Reproduction, Bangkok, Thailand: Banpot Boosiri (PI), Supawat Chutivongse (PI), Pramuan Virutamasen (CI), Chansuda Wongsrichanalai (CI), Prasarn Jimakorn (P).
*Mahidol University*, Faculty of Medicine, Siriraj Hospital, Department of Obstetrics and Gynaecology, Family Planning Research Unit, Bangkok, Thailand: Suporn Koetsawang (PI), Daungdao Rachawat (CI), Nivat Chantarakul (P).
*University of Tromsø*, Institute of Medical Biology, Tromsø, Norway: Helge Stalsberg (Reference Pathologist).
*Fred Hutchinson Cancer Research Center*, Division of Public Health Sciences, Seattle, Washington, United States of America; Co-ordinating Center: David B. Thomas (Study Co-ordinator), Elizabeth A. Noonan (statistician).
*World Health Organization*, Susan Holck, Special Programme of Research, Development and Research Training in Human Reproduction, Geneva, Switzerland.

This research received primary financial support from the Special Programme of Research, Development and Research Training in Human Reproduction, World Health Organization; and supplemental support from Contract No. N01-HD-52901 from the U.S. National Institute of Child Health and Human Development.

### References

BRESLOW, N.E. & DAY, N.E. (1980). *Statistical Methods in Cancer Research. Volume 1–The Analysis of Case–Control Studies.* International Agency for Research on Cancer: Lyon.

BRINTON, L.A., HOOVER, R., SZKLO, M. & FRAUMENI, J.F. JR. (1982). Oral contraceptives and breast cancer. *Int. J. Epidemiol.*, **11**, 316.

CANCER AND STEROID HORMONE STUDY OF THE CENTERS FOR DISEASE CONTROL AND THE NATIONAL INSTITUTE OF CHILD HEALTH AND HUMAN DEVELOPMENT (1986). Oral contraceptive use and the risk of breast cancer. *N. Engl. J. Med.*, **315**, 405.

FASAL, E. & PAFFENBARGER, R.S. JR. (1975). Oral contraceptives as related to cancer and benign lesions of the breast. *J. Natl Cancer Inst.*, **55**, 767.

HARRIS, N.V., WEISS, N.S., FRANCIS, A.M. & POLISSAR, L. (1982). Breast cancer in relation to patterns of oral contraceptive use. *Am. J. Epidemiol.*, **116**, 643.

HENNEKENS, C.H., SPEIZER, F.E., LIPNICK, R.J. & 6 others (1984). A case–control study of oral contraceptive use and breast cancer. *J. Natl Cancer Inst.*, **72**, 39.

JANERICH, D.T. & HOFF, M.B. (1982). Evidence for a crossover in breast cancer risk factors. *Am. J. Epidemiol.*, **116**, 737.

JICK, S.S., WALKER, A.M., STERGACHIS, A., & JICK, H. (1989). Oral contraceptives and breast cancer. *Br. J. Cancer*, **59**, 618.

KAY, C.R. & HANNAFORD, P.C. (1988). Breast cancer and the pill – a further report from the Royal College of General Practitioners' Oral Contraceptive Study. *Br. J. Cancer*, **58**, 675.

KELSEY, J.L., HOLFORD, T.R., WHITE, C., MAYER, E.S., KILTY, S.E. & ACHESON, R.M. (1978). Oral contraceptives and breast disease: an epidemiological study. *Am. J. Epidemiol.*, **107**, 236.

LA VECCHIA, C., DECARLI, A., FASOLI, M. & 5 others (1986). Oral contraceptives and cancers of the breast and the female genital tract. Interim results from a case–control study. *Br. J. Cancer*, **54**, 311.

LEE, N.C., ROSERO-BIXBY, L., OBERTE, M.W., GRIMALDO, C., WHATLEY, A.S. & ROVIRA, E.Z. (1987). A case–control study of breast cancer and hormonal contraception in Costa Rica. *J. Natl Cancer Inst.*, **79**, 1247.

LEES, A.W., BURNS, P.E., & GRACE, M. (1978). Oral contraceptives and breast disease in premenopausal northern Albertan women. *Int. J. Cancer*, **22**, 700.

LIPNICK, R.J., BURING, J.E., HENNEKENS, C.H. & 7 others (1986). Oral contraceptives and breast cancer. *JAMA*, **255**, 58.

MCPHERSON, K., VESSEY, M.P., NEIL, A., DOLL, R., JONES, L. & ROBERTS, M. (1987). Early oral contraceptive use and breast cancer: results of another case–control study. *Br. J. Cancer*, **56**, 653.

MEIRIK, O., LUND, E., ADAMI, H.O., BERGSTROM, R., CHRISTOFFERSON, T. & BERGSJO, P. (1986). Oral contraceptive use and breast cancer in young women. *Lancet*, **ii**, 650.

MILLER, D.R., ROSENBERG, L., KAUFMAN, D.W., STOLLY, P., WARSHAUER, E.M. & SHAPIRO, S. (1989). Breast cancer before age 45 and oral contraceptive use: new findings. *Am. J. Epidemiol.*, **129**, 269.

MUIR, C., WATERHOUSE, J.R., MACK, T., POWELL, J. & SHELAN, S. (1987). *Cancer Incidence in Five Continents. Volume V.* International Agency for Research on Cancer: Lyon.

OLSSON, H., OLSSON, M.L., MOLLER, T.R., RANSTAM. J. & HOLM. P. (1985). Oral contraceptive use and breast cancer in young women in Sweden (letter). *Lancet*, **i**, 748.

PAFFENBARGER, R.S., FASAL, E., SIMMONS, M.E. & KAMPERT, J.B. (1977). Cancer risk as related to use of oral contraceptives during fertile years. *Cancer*, **39**, 1887.

PAFFENBARGER, R.S., KAMPERT, J.B. & CHANG, H.-G. (1980). Characteristics that predict risk of breast cancer before and after the menopause. *Am. J. Epidemiol.*, **112**, 258.

PAUL, C., SKEGG, D.C.G., SPEARS, G.F.S. & KALDOR, J.M. (1986). Oral contraceptives and breast cancer: a national study. *Br. Med. J.*, **293**, 723.

PIKE, M.C., HENDERSON, B.E., CASAGRANDE, J.T., ROSARIO, I. & GRAY, G.E. (1981). Oral contraceptive use and early abortion as risk factors for breast cancer in young women. *Br. J. Cancer*, **43**, 72.

PRENTICE, R.L., O'SULLIVAN, M. & SELF, S.G. (1990). Dietary fat and breast cancer: an assessment of the epidemiologic literature in relation to the design of the woman's health trial. *Cancer Res.* (in the press).

PRENTICE, R.L. & THOMAS, D.B. (1987). On the epidemiology of oral contraceptives and disease. *Adv. Cancer Res.*, **49**, 285.

ROSENBERG, L., MILLER, D.R., KAUFMAN, D.W. & 4 others (1984). Breast cancer and oral contraceptive use. *Am. J. Epidemiol.*, **119**, 167.

ROYAL COLLEGE OF GENERAL PRACTITIONERS (1981). Breast cancer and oral contraceptives: findings in Royal College of General Practitioners' Study. *Br. Med. J.*, **282**, 2089.

SKEGG, D.C.G. (1988). Potential for bias in case–control studies of oral contraceptives and breast cancer. *Am. J. Epidemiol.*, **127**, 205.

STADEL, B.V., RUBIN, G.L., WEBSTER, L.A., SCHLESSELMAN, J.J. & WINGO, P.A. (1985). Oral contraceptives and breast cancer in young women. *Lancet*, **ii**, 970.

THOMAS, D.B. (1988). The breast. In *Symposium on Improving Safety Requirements for Contraceptive Steroids.* World Health Organization: Geneva.

U. K. NATIONAL CASE-CONTROL STUDY GROUP (1989). Oral contraceptive use and breast cancer risk in young women. *Lancet*, **i**, 973.

VESSEY, M.P., MCPHERSON, K., YEATES, D. & DOLL, R. (1982). Oral contraceptive use and abortion before first term pregnancy in relation to breast cancer risk. *Br. J. Cancer*, **45**, 327.

VESSEY, M.P., BARON, J., DOLL, R., MCPHERSON, K., & YEATES, D. (1983). Oral contraceptives and breast cancer: final report of an epidemiological study. *Br. J. Cancer*, **47**, 455.

VESSEY, M.P., MCPHERSON, K., VILLARD-MACKINTOSH, L. & YEATES, D. (1989). Oral contraceptives and breast cancer: latest findings in a large cohort study. *Br. J. Cancer*, **59**, 613.

WATERHOUSE, J., SHANMUGARATNAM, K. & POWELL, J. (eds) (1982). *Cancer Incidence in Five Continents. Volume IV.* International Agency for Research on Cancer: Lyon.

WHO COLLABORATIVE STUDY OF NEOPLASIA AND STEROID CONTRACEPTIVES (1985). Invasive cervical cancer and combined oral contraceptives. *Br. Med. J.*, **290**, 961.

WHO COLLABORATIVE STUDY OF NEOPLASIA AND STEROID CONTRACEPTIVES (1988). Edometrial cancer and combined oral contraceptives. *Int. J. Epidemiol.*, **17**, 263.

WHO COLLABORATIVE STUDY OF NEOPLASIA AND STEROID CONTRACEPTIVES (1989). Epithelial ovarian cancer and combined oral contraceptives. *Int. J. Epidemiol.* (in the press).

WORLD HEALTH ORGANIZATION (1981). *Histological Typing of Breast Tumors*, 2nd edn. WHO: Geneva.

YUAN, J.M., YU, M.C., ROSS, R.K., GAO, Y.T. & HENDERSON, B.E. (1988). Risk factors for breast cancer in Chinese women in Shanghai. *Cancer Res.*, **48**, 1949.


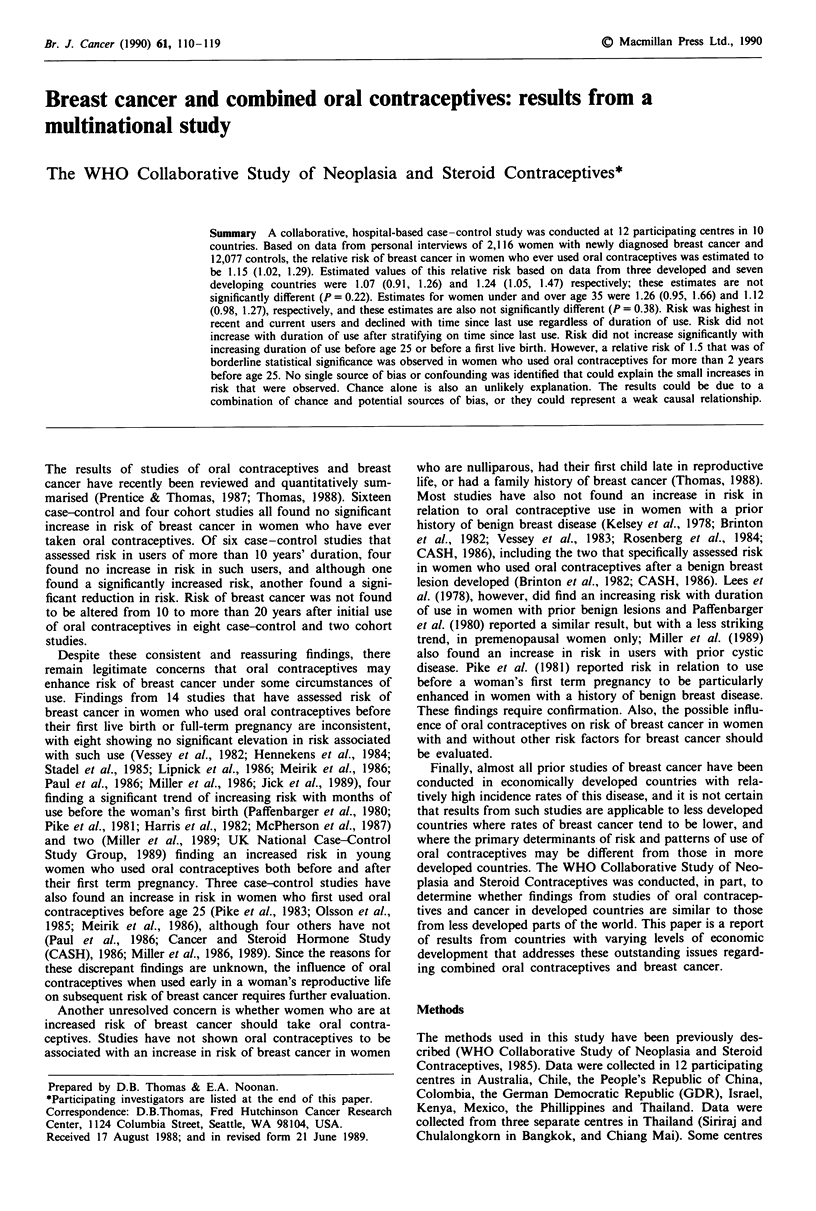

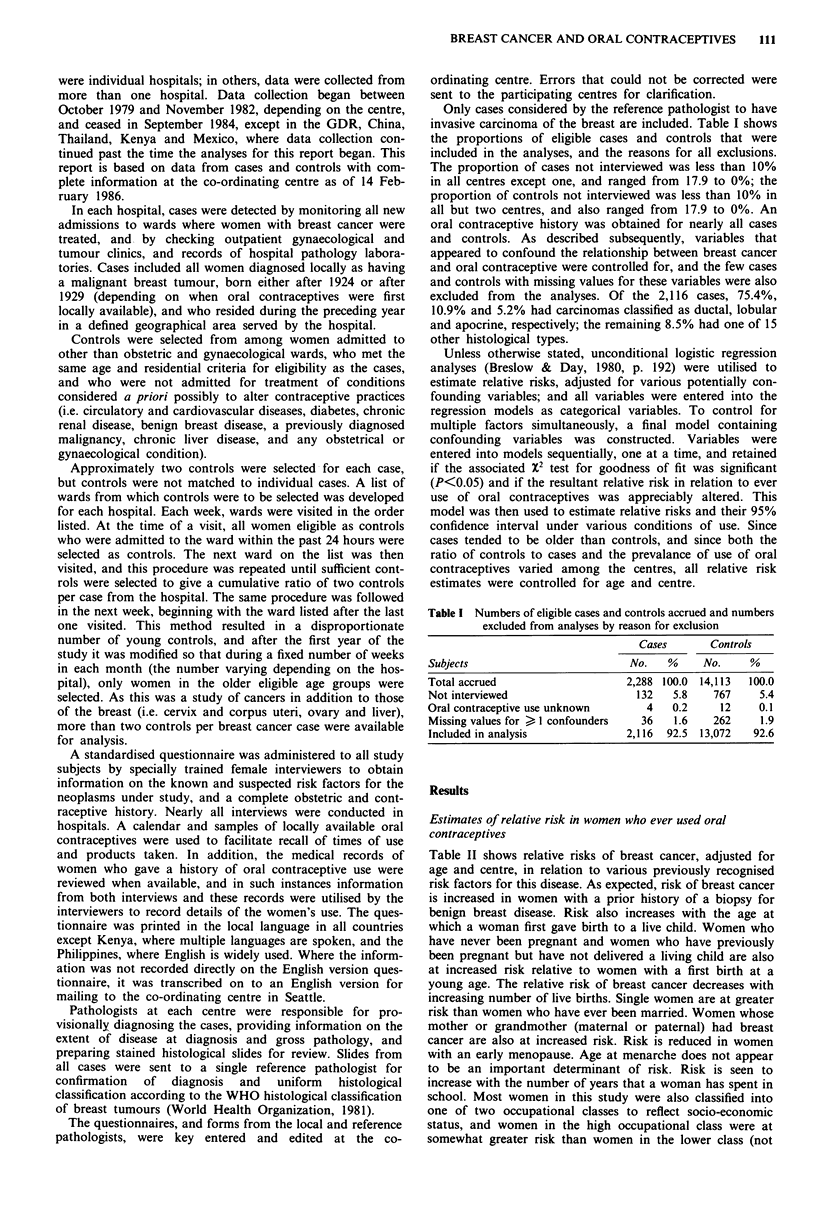

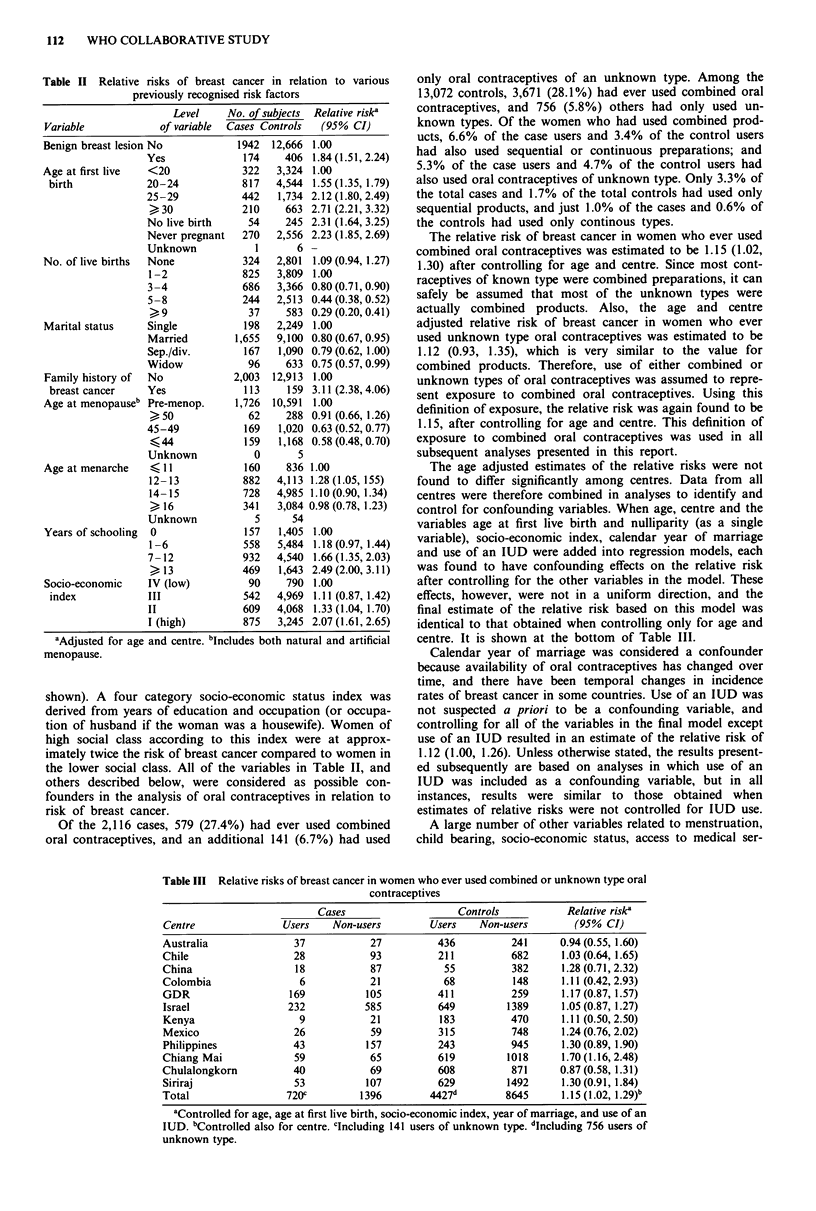

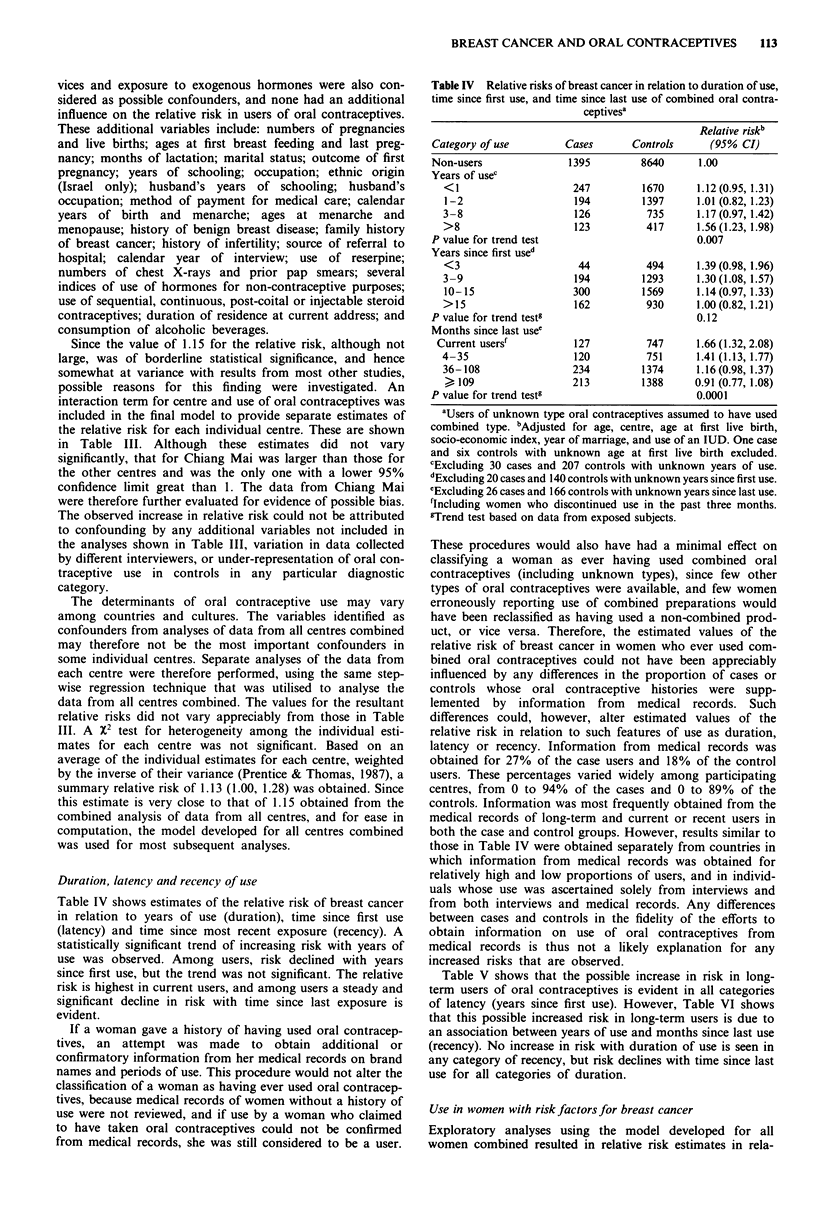

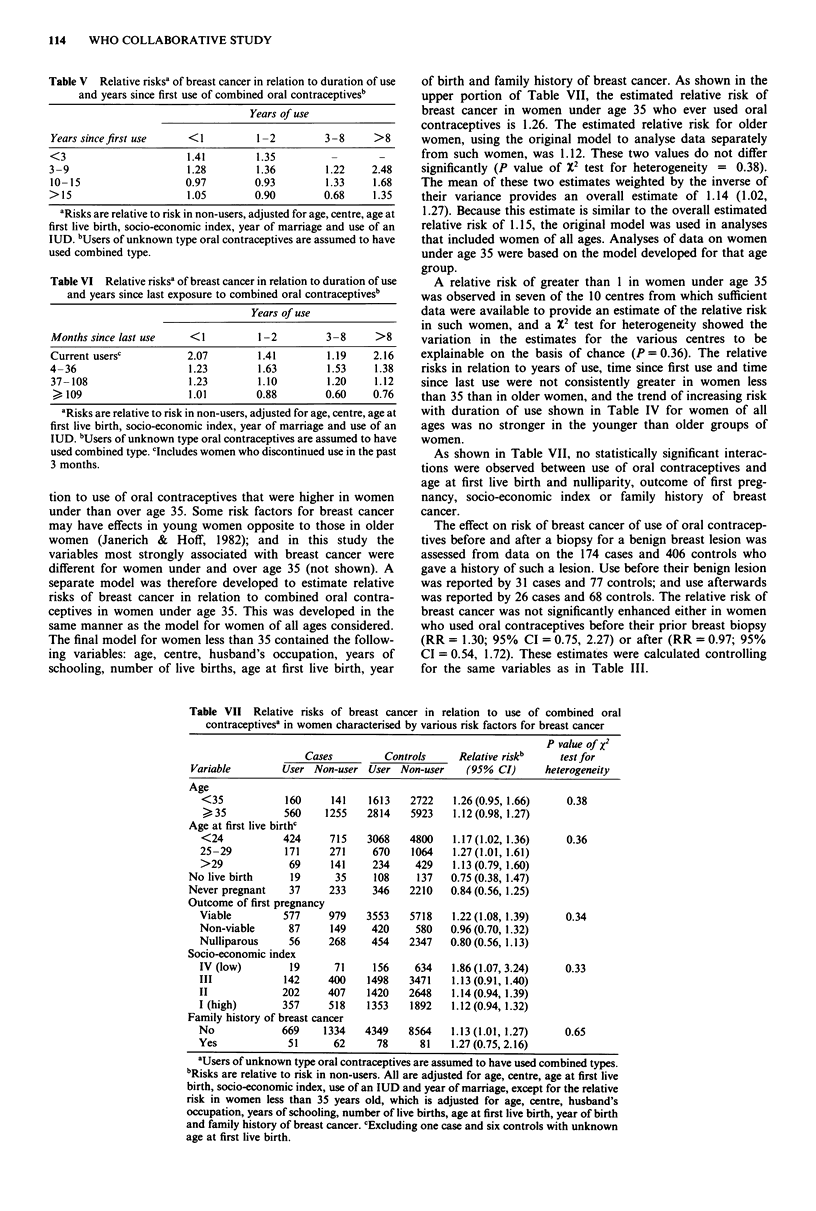

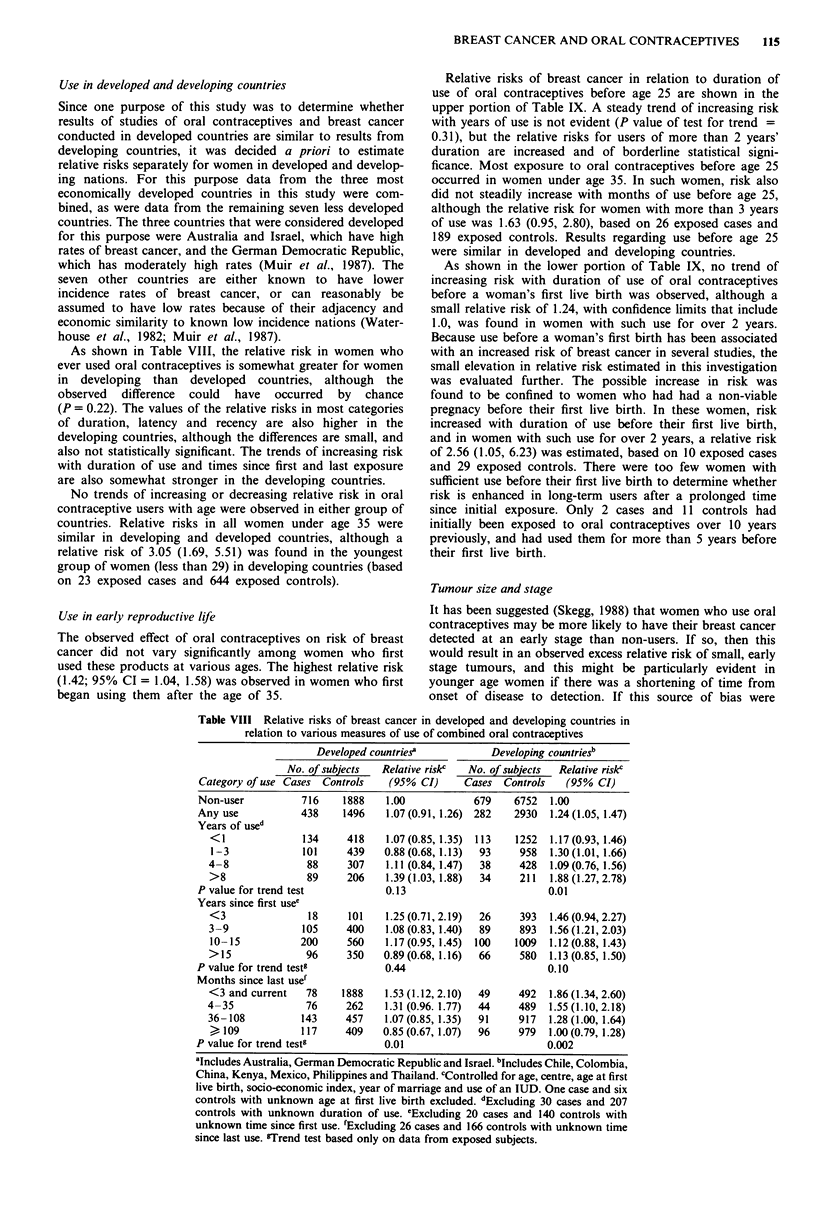

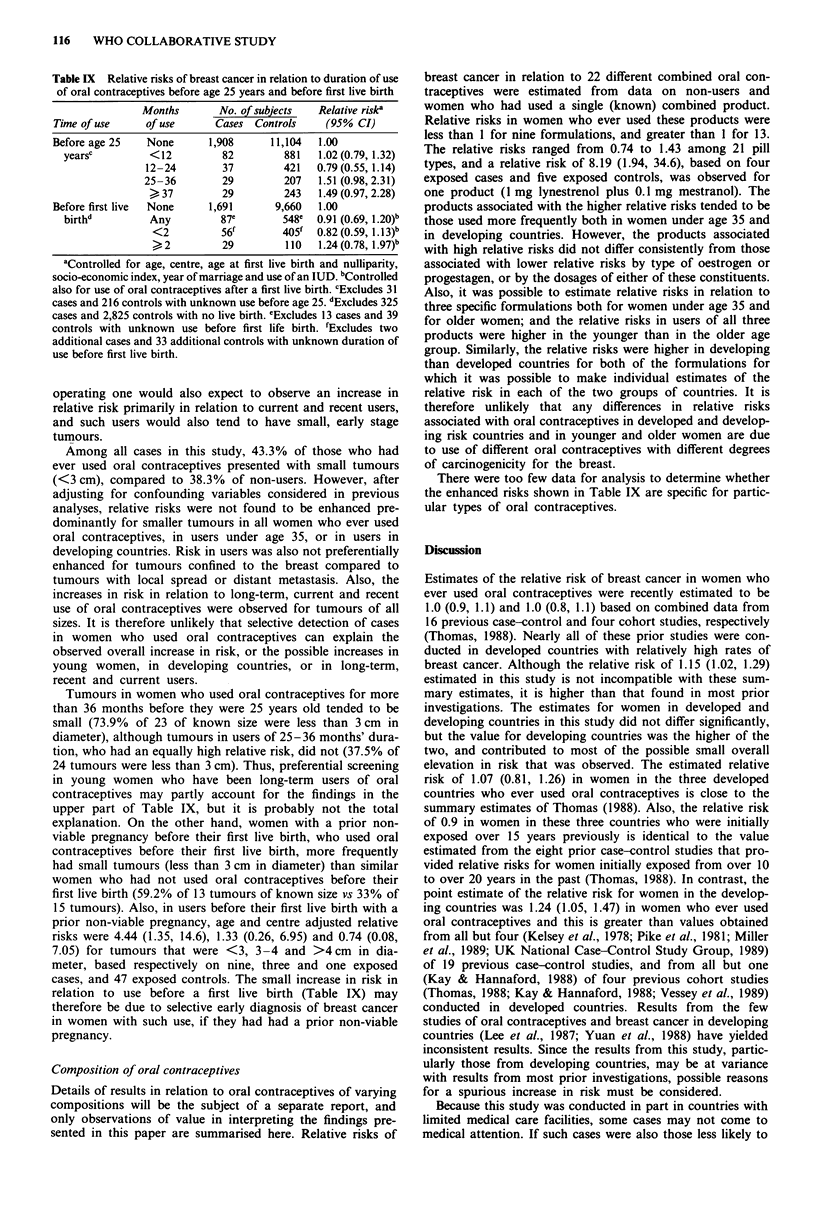

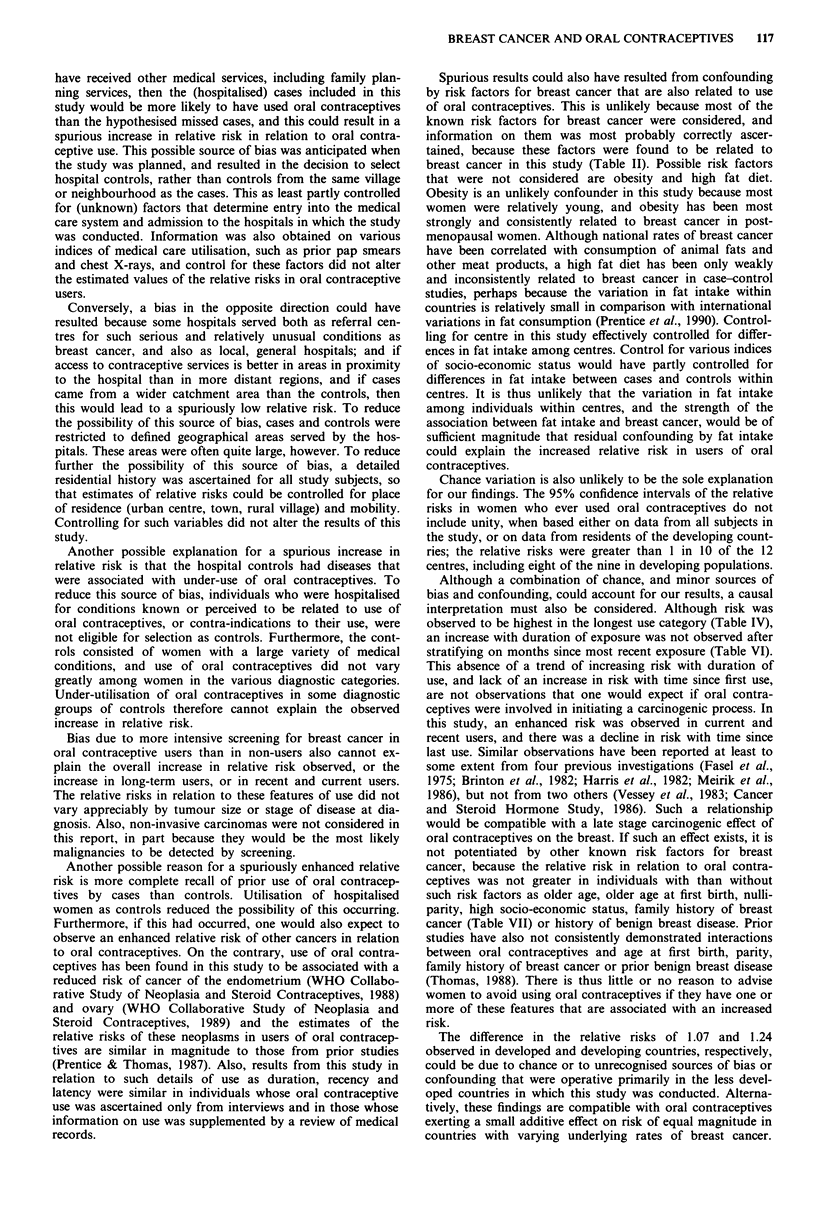

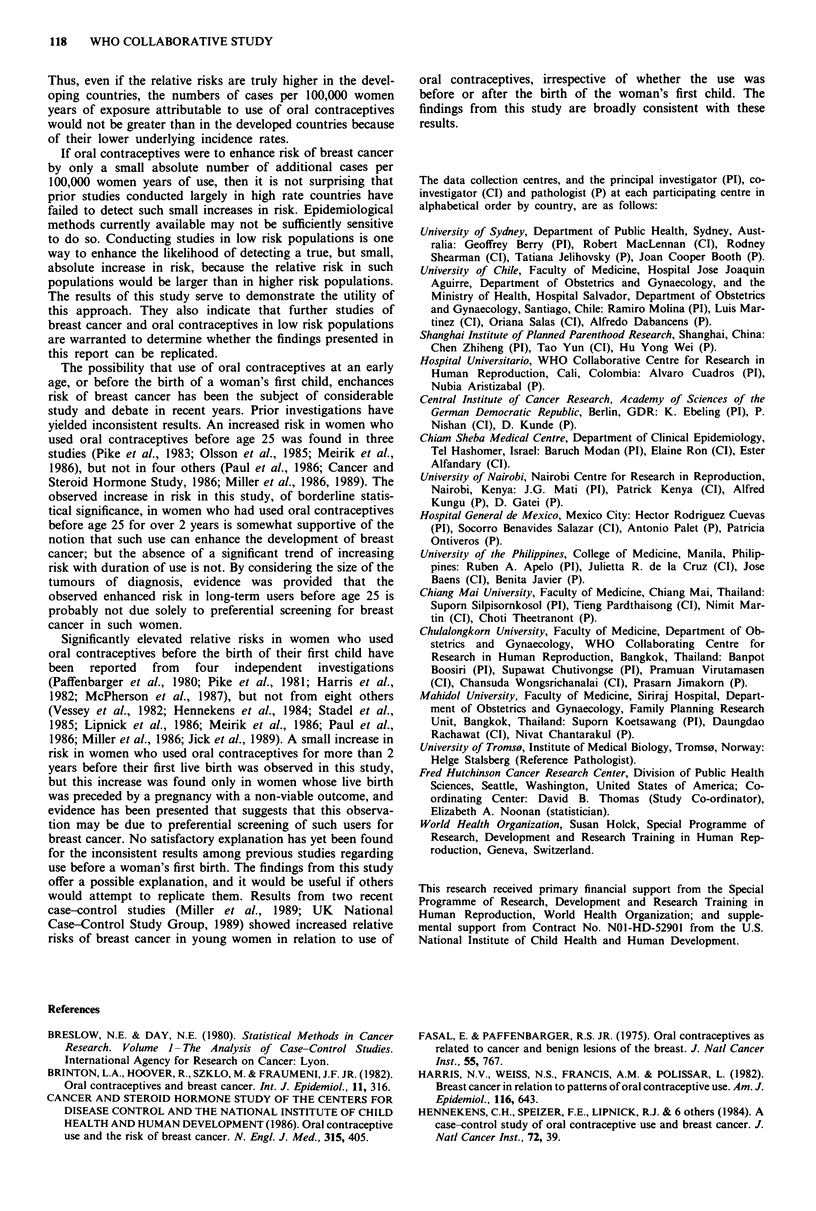

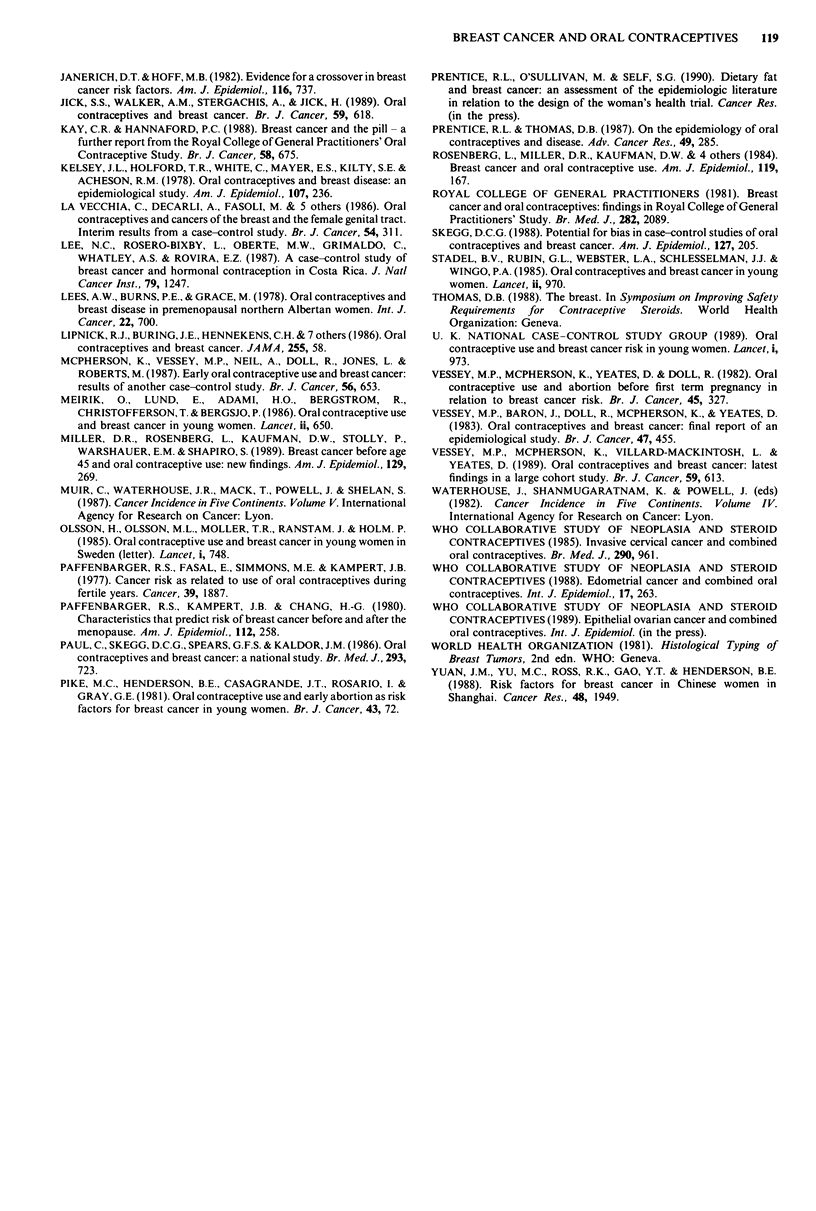


## References

[OCR_01396] Brinton L. A., Hoover R., Szklo M., Fraumeni J. F. (1982). Oral contraceptives and breast cancer.. Int J Epidemiol.

[OCR_01405] Fasal E., Paffenbarger R. S. (1975). Oral contraceptives as related to cancer and benign lesions of the breast.. J Natl Cancer Inst.

[OCR_01410] Harris N. V., Weiss N. S., Francis A. M., Polissar L. (1982). Breast cancer in relation to patterns of oral contraceptive use.. Am J Epidemiol.

[OCR_01422] Janerich D. T., Hoff M. B. (1982). Evidence for a crossover in breast cancer risk factors.. Am J Epidemiol.

[OCR_01426] Jick S. S., Walker A. M., Stergachis A., Jick H. (1989). Oral contraceptives and breast cancer.. Br J Cancer.

[OCR_01430] Kay C. R., Hannaford P. C. (1988). Breast cancer and the pill--a further report from the Royal College of General Practitioners' oral contraception study.. Br J Cancer.

[OCR_01435] Kelsey J. L., Holford T. R., White C., Mayer E. S., Kilty S. E., Acheson R. M. (1978). Oral contraceptives and breast disease. An epidemiological study.. Am J Epidemiol.

[OCR_01440] La Vecchia C., Decarli A., Fasoli M., Franceschi S., Gentile A., Negri E., Parazzini F., Tognoni G. (1986). Oral contraceptives and cancers of the breast and of the female genital tract. Interim results from a case-control study.. Br J Cancer.

[OCR_01445] Lee N. C., Rosero-Bixby L., Oberle M. W., Grimaldo C., Whatley A. S., Rovira E. Z. (1987). A case-control study of breast cancer and hormonal contraception in Costa Rica.. J Natl Cancer Inst.

[OCR_01451] Lees A. W., Burns P. E., Grace M. (1978). Oral contraceptives and breast disease in premenopausal Northern Albertan women.. Int J Cancer.

[OCR_01460] McPherson K., Vessey M. P., Neil A., Doll R., Jones L., Roberts M. (1987). Early oral contraceptive use and breast cancer: results of another case-control study.. Br J Cancer.

[OCR_01465] Meirik O., Lund E., Adami H. O., Bergström R., Christoffersen T., Bergsjö P. (1986). Oral contraceptive use and breast cancer in young women. A joint national case-control study in Sweden and Norway.. Lancet.

[OCR_01470] Miller D. R., Rosenberg L., Kaufman D. W., Stolley P., Warshauer M. E., Shapiro S. (1989). Breast cancer before age 45 and oral contraceptive use: new findings.. Am J Epidemiol.

[OCR_01481] Olsson H., Olsson M. L., Möller T. R., Ranstam J., Holm P. (1985). Oral contraceptive use and breast cancer in young women in Sweden.. Lancet.

[OCR_01486] Paffenbarger R. S., Fasal E., Simmons M. E., Kampert J. B. (1977). Cancer risk as related to use of oral contraceptives during fertile years.. Cancer.

[OCR_01491] Paffenbarger R. S., Kampert J. B., Chang H. G. (1980). Characteristics that predict risk of breast cancer before and after the menopause.. Am J Epidemiol.

[OCR_01496] Paul C., Skegg D. C., Spears G. F., Kaldor J. M. (1986). Oral contraceptives and breast cancer: a national study.. Br Med J (Clin Res Ed).

[OCR_01501] Pike M. C., Henderson B. E., Casagrande J. T., Rosario I., Gray G. E. (1981). Oral contraceptive use and early abortion as risk factors for breast cancer in young women.. Br J Cancer.

[OCR_01512] Prentice R. L., Thomas D. B. (1987). On the epidemiology of oral contraceptives and disease.. Adv Cancer Res.

[OCR_01516] Rosenberg L., Miller D. R., Kaufman D. W., Helmrich S. P., Stolley P. D., Schottenfeld D., Shapiro S. (1984). Breast cancer and oral contraceptive use.. Am J Epidemiol.

[OCR_01526] Skegg D. C. (1988). Potential for bias in case-control studies of oral contraceptives and breast cancer.. Am J Epidemiol.

[OCR_01530] Stadel B. V., Rubin G. L., Webster L. A., Schlesselman J. J., Wingo P. A. (1985). Oral contraceptives and breast cancer in young women.. Lancet.

[OCR_01555] Vessey M. P., McPherson K., Villard-Mackintosh L., Yeates D. (1989). Oral contraceptives and breast cancer: latest findings in a large cohort study.. Br J Cancer.

[OCR_01545] Vessey M. P., McPherson K., Yeates D., Doll R. (1982). Oral contraceptive use and abortion before first term pregnancy in relation to breast cancer risk.. Br J Cancer.

[OCR_01550] Vessey M., Baron J., Doll R., McPherson K., Yeates D. (1983). Oral contraceptives and breast cancer: final report of an epidemiological study.. Br J Cancer.

[OCR_01584] Yuan J. M., Yu M. C., Ross R. K., Gao Y. T., Henderson B. E. (1988). Risk factors for breast cancer in Chinese women in Shanghai.. Cancer Res.

